# A Terminal Iron Nitrilimine Complex: Accessing the Terminal Nitride through Diazo N−N Bond Cleavage

**DOI:** 10.1002/anie.201910428

**Published:** 2019-10-31

**Authors:** Sadig Aghazada, Matthias Miehlich, Julian Messelberger, Frank W. Heinemann, Dominik Munz, Karsten Meyer

**Affiliations:** ^1^ Friedrich-Alexander-Universität Erlangen-Nürnberg (FAU) Department of Chemistry and Pharmacy, General and Inorganic Chemistry Egerlandstrasse 1 91058 Erlangen Germany

**Keywords:** diazo compounds, iron, N-heterocyclic carbenes, N−N activation, terminal nitrides

## Abstract

A novel method for the N−N bond cleavage of trimethylsilyl diazomethane is reported for the synthesis of terminal nitride complexes. The lithium salt of trimethylsilyl diazomethane was used to generate a rare terminal nitrilimine transition metal complex with partially occupied d‐orbitals. This iron complex **2** was characterized by CHN combustion analysis, ^1^H and ^13^C NMR spectroscopic analysis, single‐crystal X‐ray crystallography, SQUID magnetometry, ^57^Fe Mössbauer spectroscopy, and computational analysis. The combined results suggest a high‐spin d ^6^ (S=2) electronic configuration and an allenic structure of the nitrilimine ligand. Reduction of **2** results in release of the nitrilimine ligand and formation of the iron(I) complex **3**, which was characterized by CHN combustion analysis, ^1^H NMR spectroscopic analysis, and single‐crystal X‐ray crystallography. Treatment of **2** with fluoride salts quantitatively yields the diamagnetic Fe^IV^ nitride complex **4**, with concomitant formation of cyanide and trimethylsilyl fluoride through N−N bond cleavage.

## Graphical abstract

**Chain reaction**: A fluoride salt cleaves the N–N bond of an iron trimethylsilyl nitrilimine complex and generates the terminal nitride, fluorotrimethylsilane, and cyanide. The complementary reactivity of nitrilimine and azido ligands provides a new approach for the synthesis of metal terminal nitrido complexes.

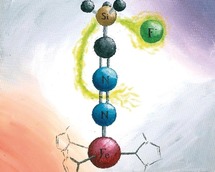

The facile release of dinitrogen renders diazo compounds powerful carbene‐transfer reagents.^1^ Hence, they find wide application in organic synthesis, including C−H bond insertion, cyclopropanation, the preparation of olefin metathesis catalysts, and even in enzyme‐catalyzed carbene‐transfer reactions.^2^ The key step in all these transformations is the generation of a transient carbene species (Scheme 1 A). Less known, diazo compounds not only serve as a carbene source, but also show nucleophilic properties at the terminal nitrogen atom, thus providing molecular systems relevant to dinitrogen activation catalyzed by the FeMo cofactor.^3^ Diazo compounds having an electron‐withdrawing group (EWG) at the carbon atom can be deprotonated to yield their lithium salts Li[(EWG)CN_2_].^4^ These ambident nucleophiles act as transient intermediates in 1,3‐dipolar cycloaddition reactions.^5^ Owing to the high reactivity of nitrilimines, coordination to transition metals is exceedingly rare, with the only examples to date being a d^0^‐configured Sc^3+^ complex having a terminal *N*‐coordinated nitrilimine reported by Chen et al. and a dinuclear iron complex having two μ_2_‐*N* bridging nitrilimine ligands described by Holland and co‐workers.^6^ There are, however, several investigations on the coordination chemistry of Li[Me_3_Si−CN_2_] with trivalent f‐elements. In all of these cases, a [1,3]‐shift of the trimethylsilyl group from the carbon to the terminal nitrogen atom was observed, which resulted in the formation of bridging isocyanotrimethylsilylamido [(Me_3_Si)NN≡C]^−^ ligands.^7^ Examples of nitrilimine N−N bond cleavage with nitride formation are hitherto unknown.

**Scheme 1:**
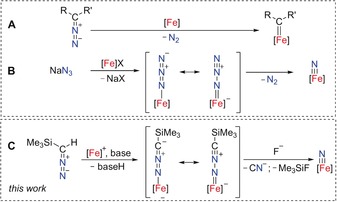
A) Diazo compounds as carbene‐transfer reagents, B) azides as nitride‐transfer reagents, and C) trimethylsilyl diazomethane as a nitride‐transfer reagent via an intermediate nitrilimine complex.

The nitrilimine anion is isoelectronic to the azido ligand, N_3_^−^, which is a common, but—due to poor solubility—often problematic precursor to generate terminal metal nitrides upon release of dinitrogen (Scheme 1 B).^8^ The investigation of terminal iron nitride complexes promises novel approaches for dinitrogen valorization.^9^ Yet, the synthesis of terminal nitride complexes remains a challenge in coordination chemistry. In our efforts to develop novel synthetic routes to nitride intermediates, we investigated terminal *N*‐bound nitrilimine ligands as possible starting materials. In this approach (Scheme 1 C) the N−N bond cleavage is analogous to the commonly observed C−N bond cleavage reaction of diazo compounds to give carbene complexes (Scheme 1 A).

As a precursor, we chose the iron(II) complex **1**, which has the *N*‐anchored *tris*‐*N*‐heterocyclic carbene ligand TIMEN^mes^ (tris[2‐(3‐mesitylimidazolin‐2‐ylidene)ethyl]amine) sporting bulky mesityl groups at the N‐heterocyclic carbene (NHC). This ligand provides sufficient steric bulk to prevent the formation of undesired dinuclear complexes.9b, 10 Indeed, reaction of the trimethylsilyl diazomethane lithium salt Li[Me_3_Si−CN_2_] with the Fe^II^ *tris*‐carbene complex [(TIMEN^mes^)FeCl](BPh_4_) (**1**) in tetrahydrofuran at −100 °C resulted in an orange‐colored solution that intensified its color upon warming to ambient temperature (Scheme 2). After workup, the orange, crystalline complex [(TIMEN^mes^)Fe(N_2_CSiMe_3_)](BPh_4_) (**2**) was obtained in 91 % yield. The paramagnetically shifted and broadened ^1^H NMR signals of **2** are in accordance with a complex of trigonal symmetry; the solid‐state infrared (IR) spectrum of **2** is reminiscent of the spectrum of its azido analogue [(TIMEN^mes^)Fe(N_3_)]^+^ (ν_as_(N_3_^−^)=2094 cm^−1^) and exhibits a strong nitrilimine absorption band centered at 2068 cm^−1^.9b

**Scheme 2:**
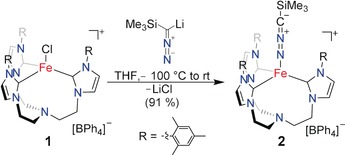
Synthesis of nitrilimine complex **2. Note**: Li[Me_3_Si−CN_2_] is pyrophoric and should be handled with care.

Orange single crystals suitable for X‐ray diffraction (SC‐XRD) analysis were obtained at room temperature by diffusion of diethyl ether or pentane vapor into solutions of **2** in tetrahydrofuran (Figure 1). In the molecular structure, the three carbene entities and the trimethylsilyl nitrilimine provide a pseudo‐tetrahedral, approximate *C*_3_‐symmetric coordination environment for the central iron metal ion (Fe: 0.615(2) Å above the plane defined by the three carbene carbon atoms; *τ*_4_=0.95; *τ*_T_=1; *τ*_trig.pyr._=0.85).^11^

**Figure 1:**
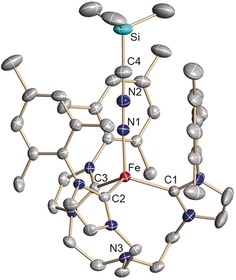
Solid‐state molecular structure of the iron–nitrilimine complex [(TIMEN^mes^)Fe(N_2_CSiMe_3_)](BPh_4_) (**2**) in crystals of **2⋅**3.5 THF. Thermal ellipsoids are shown with 50 % probability; H atoms, the (BPh_4_)^−^ anion, and co‐crystallized solvent molecules are omitted for clarity. Selected bond lengths (Å) and angles (°): Fe‐C1 2.095(3); Fe‐C2 2.091(3); Fe‐C3 2.100(3); Fe‐N1 1.922(3); N1‐N2 1.199(4); N2‐C4 1.200(4); C4‐Si 1.775(4); Fe‐N3 3.321(3); C1‐Fe‐C2 117.8(2); C2‐Fe‐C3 108.6(2); C2‐Fe‐C3 108.7(2); Fe‐N1‐N2 177.0(2); N1‐N2‐C4 179.7(4); N2‐C4‐Si 179.3(4).

To the best of our knowledge, this is a rare example of a terminal nitrilimine ligand coordinated to a transition metal (vide supra).^6^ The nitrilimine ligand in **2** is nearly linear [∡(Fe‐N_a_‐N_b_) 177.0(2)°; ∡(N_a_‐N_b_‐C) 179.7(4)°; ∡(N_b_‐C‐Si) 179.3(4)°] with N−N and N−C bond lengths of 1.199(4) Å and 1.200(4) Å, respectively. These bond lengths indicate that the electronic structure of the nitrilimine ligand is best described in an allenic rather than a propargylic resonance structure.^12^ The average Fe−C_carbene_ bond length [2.095(3) Å] is comparable to that of the azido analogue [2.108(3) Å].9b

The ^57^Fe Mössbauer spectrum of **2** at 77 K and zero field (Figure 2 A) shows a single asymmetric quadrupole doublet with an isomer shift of *δ*=0.65 mm s^−1^ and a quadrupole splitting of Δ*E*_Q_=1.97 mm s^−1^. These values are indicative of a high‐spin iron(II) metal center and are similar to the values reported for the azido complex (*δ*=0.69 mm s^−1^; Δ*E*_Q_=2.27 mm s^−1^).9b According to the SQUID measurements (Figure 2 B), complex **2** possesses an effective magnetic moment, *μ*_eff_, of 5.19 *μ*_B_ at 300 K (Evans method: *μ*_eff_=5.01 μ_B_), which is higher than the spin‐only value of 4.90 expected for an *S*=2 high‐spin d^6^ Fe^II^ complex. The magnetic moment increases slightly at lower temperatures likely due to intermolecular coupling, and decreases below 50 K to reach *μ*_eff_ of 4.1 μ_B_ at 2 K. The magnetization measurements at varying magnetic fields were fitted with the isotropic *g*_iso_ value of 2.19, resulting in the zero‐field splitting (ZFS) *D*=−5.57 cm^−1^ and the rhombic ZFS *E=*0 parameters (Figure 2 B, inset). Solid and frozen THF solution samples of complex **2** are EPR‐silent (at 300 and 10 K, in perpendicular mode X‐Band), thus supporting the integer spin state assignment.

**Figure 2:**
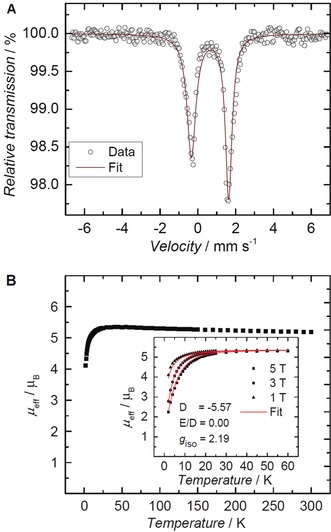
A) Zero‐field ^57^Fe Mössbauer spectrum of a solid sample of **2** collected at 77 K. B) Plot of the effective magnetic moment, *μ*_eff_ , versus temperature, from temperature‐dependent SQUID magnetization measurements of **2** at 1 T (*μ*_eff_=5.19 μ_B_, 300 K). Inset: variable‐field variable‐temperature magnetization measurements at 1, 3, and 5 T. Simulation yields parameters *g*_iso_=2.19, *D*=−5.57 cm^−1^, and *E/D*=0.

In order to further elucidate the electronic structure of **2**, quantum chemical calculations were performed. Geometry optimization in the quintet state by density functional theory (DFT; BP86‐D3BJ/def2‐TZVPP//BP86‐D3BJ/def2‐SVP) afforded geometric parameters in excellent agreement with the solid‐state structure. Also, the calculated IR stretching frequency (2119 cm^−1^), as well as the Mössbauer isomer shift (*δ*=0.37 mm s^−1^) and quadrupole splitting (Δ*E*_Q_=2.54 mm s^−1^) match the experimental data (cf. Figure 2 A). Subsequent CASSCF/NEVPT2 calculations with truncated mesityl substituents suggest a quintet d^6^ electronic ground state, with a 2+1+2 ligand field splitting and a doubly occupied d${}_{x{}^{2}-y{}^{2}}$ orbital (Figure 3). The energies of the doubly degenerate nitrilimine π‐orbitals are of the same order of magnitude as the metal d‐orbitals (Figure S20). Furthermore, the calculations corroborate an allenic rather than propargylic resonance structure, as indicated by the Mayer bond orders of 0.9 for Fe−N, 2.2 for N−N, and 2.4 for N−C as well as the accumulation of negative partial charge at the carbon atom (−0.3 a.u.).

**Figure 3:**
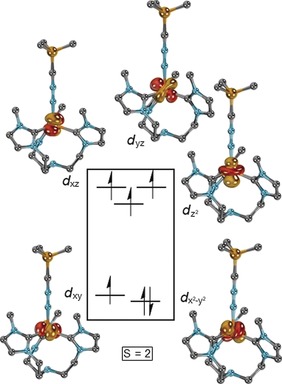
Iron d‐orbital splitting of the truncated cation of **2**, as predicted from CASSCF(10,9) calculations.

The cyclic voltammogram of **2** reveals a quasi‐reversible oxidation *E*_1/2_ at −0.71 V and an apparently irreversible reduction *E*_p,c_ at −2.6 V (vs. FeCp_2_^+^/FeCp_2_) (Figure 4, and see the Supporting Information for a detailed discussion). The anodic current at −1.78 V is related to the oxidation of reduced **2^red^** back to **2**.

**Figure 4:**
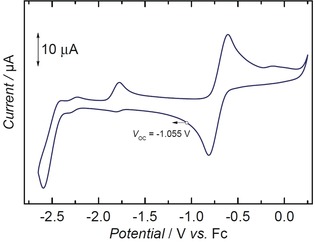
Cyclic voltammogram of **2**, measured in THF (0.1 m NBu_4_PF_6_), referenced vs. the Fc/Fc^+^ couple, with a scan rate of 100 mV s^−1^.

Whereas our attempts to isolate oxidized **2** were unsuccessful, reduction with 1.2 equivalents of KC_8_ (Scheme 3) yielded a dark red solution. The ^1^H NMR spectroscopic analysis of the crude reaction mixture revealed loss of the axial nitrilimine ligand and formation of the *C*_3_‐symmetric complex [(TIMEN^mes^)Fe](BPh_4_) (**3**).7a This monovalent complex **3** could also be synthesized independently by reduction of **1** with Na/Hg. The SC‐XRD analysis of **3** confirmed the formation of the iron(I) complex [(TIMEN^mes^)Fe](BPh_4_). According to the crystallographic analysis, the iron center is in a trigonal‐pyramidal coordination environment (*τ*_4_=0.86),^11^ with an average Fe−C_carbene_ bond distance of 2.017 Å and an Fe−N bond distance of 2.270(3) Å (Figure 5). Further, ^1^H NMR spectroscopic analysis corroborated that Li[Me_3_Si‐CN_2_] does not react with **3**. This lack of reactivity is peculiar as azidotrimethylsilane (Me_3_Si)N_3_, which is isoelectronic to [(SiMe_3_)CN_2_]^−^, is known to react swiftly with **3**, providing the Fe^II^ azide complex with concomitant release of hexamethyldisilane.9b

**Figure 5:**
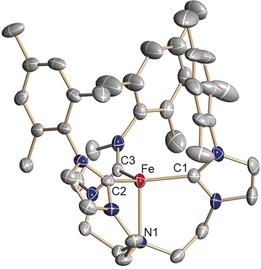
Solid‐state molecular structure of the cationic unit of [(TIMEN^mes^)Fe](BPh_4_) (**3**) in crystals of **3⋅**2 THF⋅0.5 Et_2_O. Thermal ellipsoids are shown at 50 % probability; H atoms, the (BPh_4_)^−^ anion, and co‐crystallized solvent molecules are omitted for clarity. Selected bond lengths (Å) and angles (°): Fe‐C1 2.025(3); Fe‐C2 2.022(3); Fe‐C3 2.004(3); Fe‐N1 2.270(3); C1‐Fe‐C2 120.0(2); C2‐Fe‐C3 117.9(2); C2‐Fe‐C3 119.4(2); average (N1‐Fe‐C) 95.6(2).

**Scheme 3:**
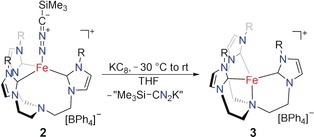
Reduction of **2** leads to the release of the nitrilimine ligand and formation of the Fe^I^ complex **3**.

Encouraged by the electronic analogy between the terminal nitrilimine and azido ligands, we also tested the transformation of the nitrilimine to the terminal nitride. Since irradiating solutions of **2** did not yield the expected N−N bond cleavage, we decided to drive this transformation via the abstraction of the trimethylsilyl group.^13^ Indeed, treating a THF solution of complex **2** with tetrabutylammonium fluoride at −30 °C resulted in an instantaneous color change to intense purple (Scheme 4). The ^1^H and ^13^C NMR spectra were in excellent agreement with the previously reported data of the iron(IV) nitride, [(TIMEN^mes^)Fe(N)](BPh_4_) (**4**), and are proof of the unprecedented, quantitative and clean conversion of **2** to the diamagnetic nitrido complex (**4**).9b The ^1^H and ^13^C NMR spectroscopic analysis revealed the concomitant formation of Me_3_SiF and NBu_4_CN (Figures S11 and S12). For structural proof, complex **4** was recrystallized and SC‐XRD analysis unambiguously confirmed the formation of the terminal iron nitride complex (see the Supporting Information for SC‐XRD details). This N−N bond cleavage of a diazo compound is unique, with the only comparable reactivity found for pincer‐type iron complexes reported by Chirik et al. to give iron nitrile and aldimine complexes.^14^ However, for those complexes, a mechanism involving a second diazo molecule leading to an intermediate metallo‐heterocyclic azine was proposed. Other reports on the N−N bond cleavage of diazo compounds relate to reductive processes via hydrogenation.^15^ We conclude that the fluoride treatment of a trimethylsilyl nitrilimine coordinated complex provides a new entry into metal–nitrido synthesis under exceptionally mild reaction conditions. Additionally, we note that the high solubility of Li[Me_3_Si−CN_2_] offers advantages compared to most common azide salts that often suffer from non‐quantitative conversion to the desired azido complex in organic solutions.

**Scheme 4:**
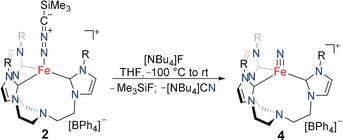
Nitrilimine N−N bond cleavage in complex **2** via trimethylsilyl group abstraction leads to the iron nitride complex **4**.

In conclusion, we here report the isolation of the unprecedented terminal nitrilimine transition metal complex **2**. Reduction of the iron(II) nitrilimine **2** leads to release of the nitrilimine ligand with formation of the iron(I) complex **3**. Remarkably, treatment of **2** with a fluoride salt forms the terminal iron(IV) nitride **4** under mild reaction conditions. This work demonstrates the complementary reactivity of nitrilimine and azido ligands and introduces a new synthetic approach for the synthesis of metal–nitrido complexes.

## Conflict of interest

The authors declare no conflict of interest.

## Supporting information

As a service to our authors and readers, this journal provides supporting information supplied by the authors. Such materials are peer reviewed and may be re‐organized for online delivery, but are not copy‐edited or typeset. Technical support issues arising from supporting information (other than missing files) should be addressed to the authors.

Supplementary
