# Supplementary material for: A Terminal Iron Nitrilimine Complex: Accessing the Terminal Nitride through Diazo N−N Bond Cleavage
Source: Angew Chem Int Ed Engl. 2019 Oct 31;58(51):18547–51. doi: 10.1002/anie.201910428 (PMC6916540; doi:10.1002/anie.201910428)
Supplement: Supplementary file 1 — Supplementary [file ANIE-58-18547-s001.pdf]

Angewandte Chemie
Eine Zeitschrift der Gesellschaft Deutscher Chemiker

Supporting Information

A Terminal Iron Nitrilimine Complex: Accessing the Terminal Nitride through Diazo N–N Bond Cleavage

Sadig Aghazada, Matthias Miehlich, Julian Messelberger, Frank W. Heinemann, Dominik Munz, and Karsten Meyer**

anie_201910428_sm_miscellaneous_information.pdf

Contents

General Information S2
Synthesis S3
Spectroscopic and Electrochemical Characterization of **2** S8
Reduction of Iron Nitrilimine Complex **2** to Iron Complex **3** S12
N–N Bond Cleavage of Iron Nitrilimine Complex **2** to Iron Nitrido Complex S15
XRD Analyses S18
Computational Details S26
References S33

General Information

All air and moisture-sensitive experiments were conducted under dry nitrogen atmosphere using a Schlenk line or an MBraun glovebox. Anhydrous iron(II) chloride was obtained from Sigma-Aldrich and used as received. Solvents were purified using a two-column solid-state purification system (Glasscontour System, Irvine, CA) and stored over activated 4 Å molecular sieves. Deuterated solvents were obtained packaged under Argon and were stored over activated molecular sieves. Anhydrous NBu_4F,[1] [TIMEN^{mes}FeCl]Cl,[2] and the lithium salt of trimethylsilyl diazomethane[3] were prepared according to the literature procedures.

^{1}H and ^{13}C NMR spectra were recorded on JEOL 270 and 400 MHz instruments with a probe temperature of 23 ºC at operating frequencies of 269.714 MHz and 400.178 MHz for ^{1}H, and 67.82 MHz and 100.624 MHz for ^{13}C measurements. The solvent residual signals were used as internal reference for the reported ^{1}H NMR and ^{13}C NMR chemical shifts. Electronic absorption spectra were collected with a UV-vis-NIR Spectrophotometer (Shimadzu UV-3600) and infrared spectra were recorded on the solid sample in KBr pellets using a Shimadzu IR Prestige-21 spectrometer.

The zero-field ^{57}Fe Mößbauer spectrum was collected on a WissEl spectrometer (MRG-500) at 77 K with alternating constant acceleration and 0.8 GBq ^{57}Co/Rh γ-source. The minimum experimental line width was 0.24 mms^{-1}. The temperature of the samples was controlled by an MBBC-HE0106 MÖSSBAUER He/N_2 cryostat within an accuracy of ±0.3 K. Isomer shifts are quoted relative to iron metal at 300 K. Mößbauer data were analyzed and simulated using the software "mcal" and "mf" written by E. Bill (mail: ebill@gwdg.de, MPI for Chemical Energy Conversion, Mülheim an der Ruhr, Germany).

Magnetism data of crystalline, finely powdered samples (17 – 24 mg) in a polycarbonate gel capsule, were recorded with a Quantum Design MPMS3 SQUID magnetometer. DC magnetization data were collected in the temperature range 2 – 300 K with an applied DC field of 1 T. Values of the magnetic susceptibility were corrected for core diamagnetism of the sample estimated using tabulated Pascal's constants.[4] Magnetic susceptibility data were analyzed and simulated using the software "julX2" written by E. Bill (mail: ebill@gwdg.de, MPI for Chemical Energy Conversion, Mülheim an der Ruhr, Germany).

Electron paramagnetic resonance spectra were recorded on a JEOL continuous wave spectrometer JES-FA200, equipped with an X-band Gunn diode oscillator bridge, a cylindrical mode cavity, and a helium cryostat. The sample was freshly dissolved in dry THF to match an approximately 1 mM concentration and placed in an air-tight J. Young quartz EPR tube. Immediately after dissolution, the sample (in the EPR tube) was taken out of the glovebox, frozen in liquid nitrogen, and kept frozen until measured. Analysis and simulation of the data was performed using the software "eview" and "esim" written by E. Bill (mail: ebill@gwdg.de, MPI for Chemical Energy Conversion, Mülheim an der Ruhr, Germany).

Elemental analyses were conducted using Euro EA 3000 (Euro Vector) and EA 1108 (Carlo-Erba) combustion analyzers available at the Chair of Inorganic and General Chemistry at FAU Erlangen-Nürnberg.

Synthesis

***[TIMEN^{mes}FeCl](BPh$_4$)* (1)**

A solution of $NaBPh_4$ (230 mg, 0.67 mmol, 1.05 equiv.) in 6 mL THF was added to a slurry of [TIMEN^{mes}FeCl]Cl (500 mg, 0.64 mmol, 1 equiv.) in 8 mL of THF. The reaction mixture was filtered after stirring for 30 min and the colorless filtrate was concentrated to *ca.* 2–3 mL. The product precipitated after addition of *ca.* 5 mL of diethyl ether. The precipitate was isolated by filtration and dried under high vacuum to afford a colorless solid (Yield: 540 mg, 79%).

Elemental Analysis (%): Calculated for $C_{66}H_{71}FeN_7BCl$: C 74.47, H 6.72, N 9.22. Found: C 74.54, H 6.60, N 8.73.

^{1}H NMR (400 MHz, DMSO-d_6): δ 75.96 (s, 3H), 24.66 (s, 3H), 20.79 (s, 10H), 18.83 (s, 2H), 9.46 (s, 3H), 7.19 (s, 8H), 6.92 (t, J = 7.2 Hz, 8H), 6.78 (t, J = 6.9 Hz, 4H), 5.04 (s, 3H), 4.79 (s, 9H), -0.28 (s, 9H), -5.15 (s, 9H) ppm.

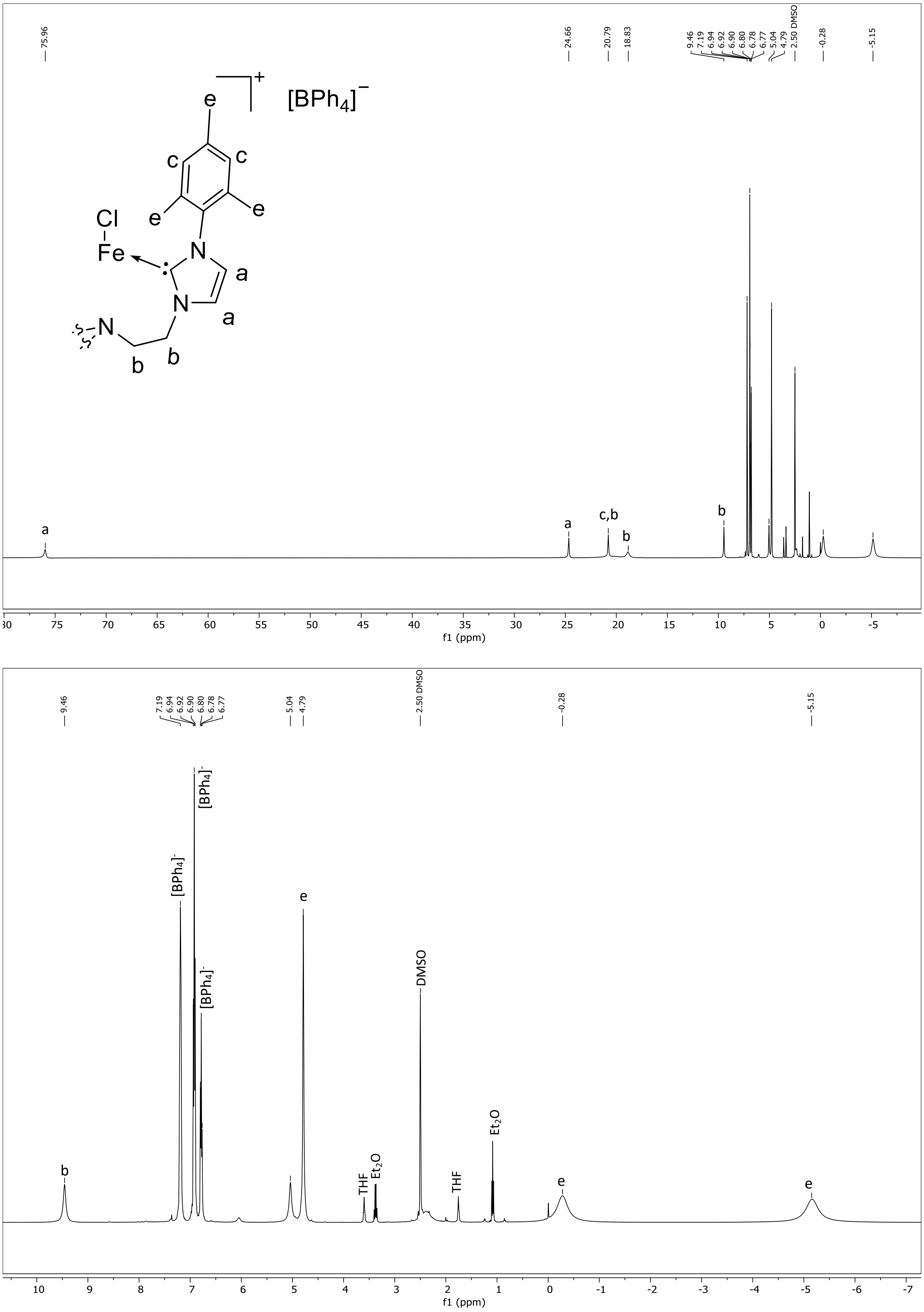

Figure S1: ^{1}H NMR of **1** in DMSO-d_6.

***[TIMEN^{mes}Fe(N$_2$C-SiMe$_3$)](BPh$_4$)* (2)**

White [TIMEN^{mes}FeCl](BPh$_4$) (200 mg, 0.188 mmol) was dissolved in 5 mL of THF and cooled to −100 °C in a pre-cooled coldwell. Subsequently, a solution of Li[Me$_3$Si-CN$_2$] (24.8 mg, 0.207 mmol, 1.1 equiv.) in 4 mL of THF was added dropwise resulting in instant color change to form an orange cloudy solution. The reaction mixture was slowly warmed to ambient temperature and stirred for an additional hour. The mixture was filtered, and the filtrate concentrated to *ca.* 3 mL. The orange product crystallized upon storage at −30 °C overnight and after collection, it was dried under high vacuum (140 mg). Et$_2$O vapor diffusion into the supernatant THF solution afforded further 55 mg of the product (overall yield: 195 mg, 91%).

Elemental Analysis (%): Calculated for $C_{70}H_{80}FeN_9BSi$: C 73.60, H 7.06, N 11.04. Found: C 73.71, H 7.24, N 11.17%.

^{1}H NMR (270 MHz, THF-*d*$_8$): δ 85.83 (s, 3H), 33.81 (br., 6H), 28.89 (s, 3H), 28.22 (s, 8H), 19.67 (s, 3H), 19.01 (s, 3H), 8.48 (s, 8H), 7.54 (s, 8H), 7.21 (s, 4H), 4.91 (s, 9H), 3.2 – 1.8 (br., 14H), -14.64 (s, 9H) ppm.

^{13}C NMR (101 MHz, THF-*d*$_8$): δ 184.96, 175.40, 167.26, 166.77, 166.27, 165.78, 146.12, 143.53, 139.26, 138.95, 127.27, 123.25, 112.18, 68.23, 67.98, 67.85, 66.31, 58.02, 26.37, 24.91, 23.86, 15.70, 11.71 ppm.

IR in KBr pellet (cm^{-1}): 2068, 1482, 1440, 1389, 1237, 1125, 1029, 840, 737.

UV-Vis λ(nm) / ε (10^{-3} M^{-1}cm^{-1}): 446 / 4.26; 496(sh) / 1.98.

The solution phase magnetic moment of 5.01 μ_B, was estimated using the Evans method. For this, 9.5 mg of **2** were dissolved in 8.88 g of a solvent mixture of THF : THF-*d*$_8$: SiMe$_4$ (10 mL : 2 mL : 1 mL). A capillary was filled with the solution of **2** and sealed. An NMR tube containing the sample capillary was filled with the same solvent mixture. Measurements were conducted at 23 °C with a 400 MHz instrument. The shift of the SiMe$_4$ ^{1}H signal was used for the magnetic moment calculations.

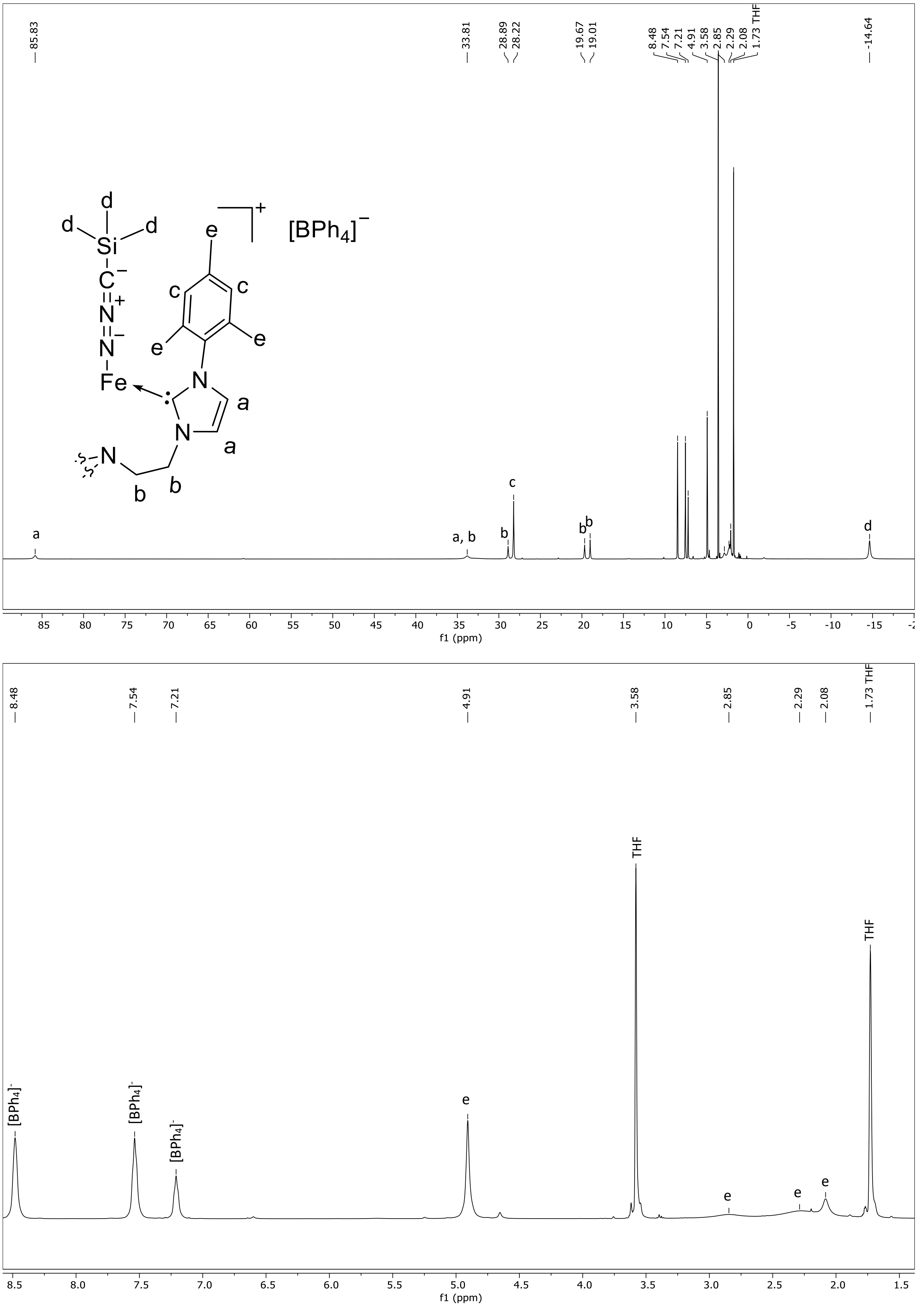

Figure S2. ^{1}H NMR spectrum of **2** in THF-d_8.

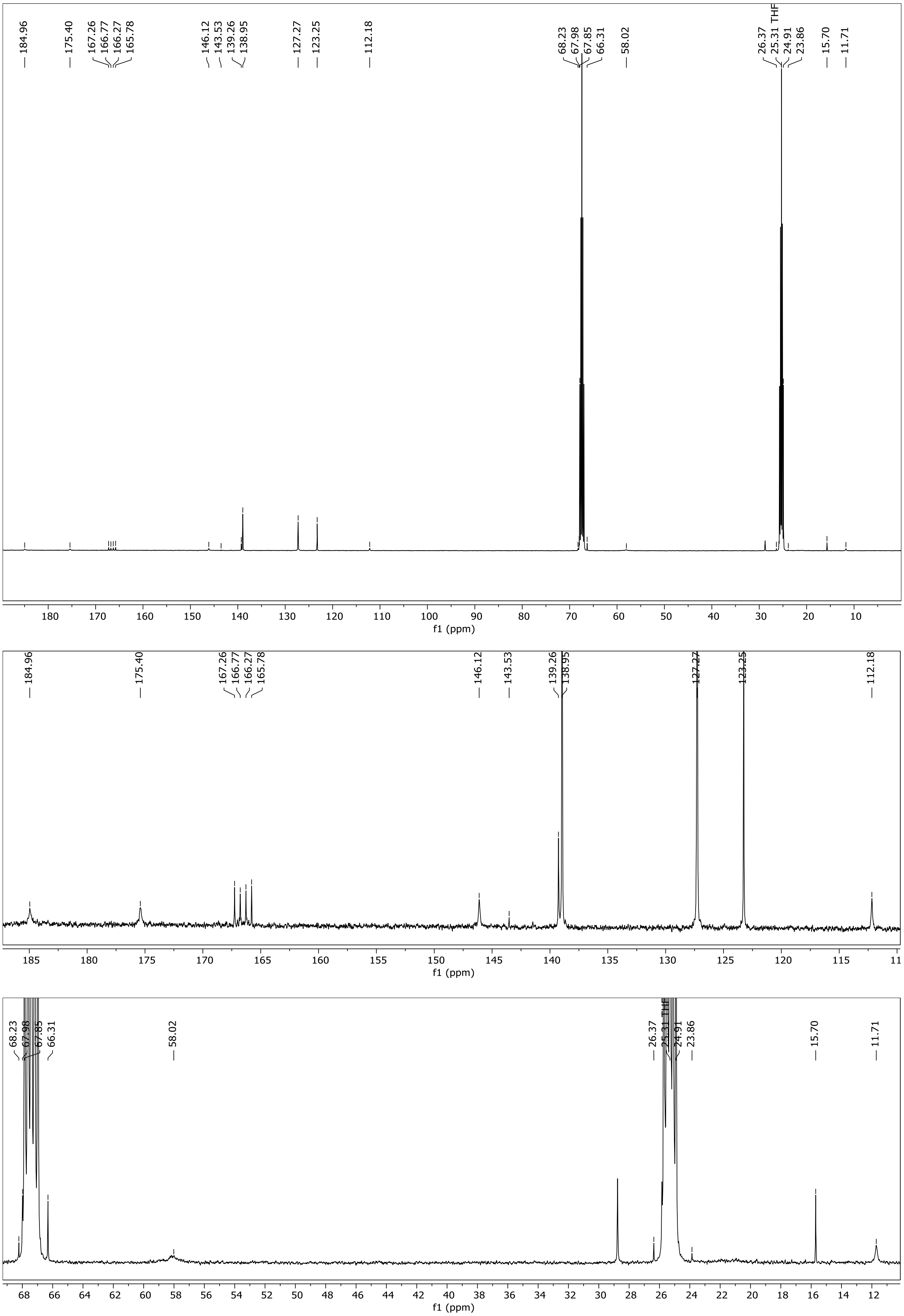

Figure S3. ^{13}C NMR spectrum of **2** in THF-d_8.

Spectroscopic and Electrochemical Characterization of 2

Infrared vibrational analysis

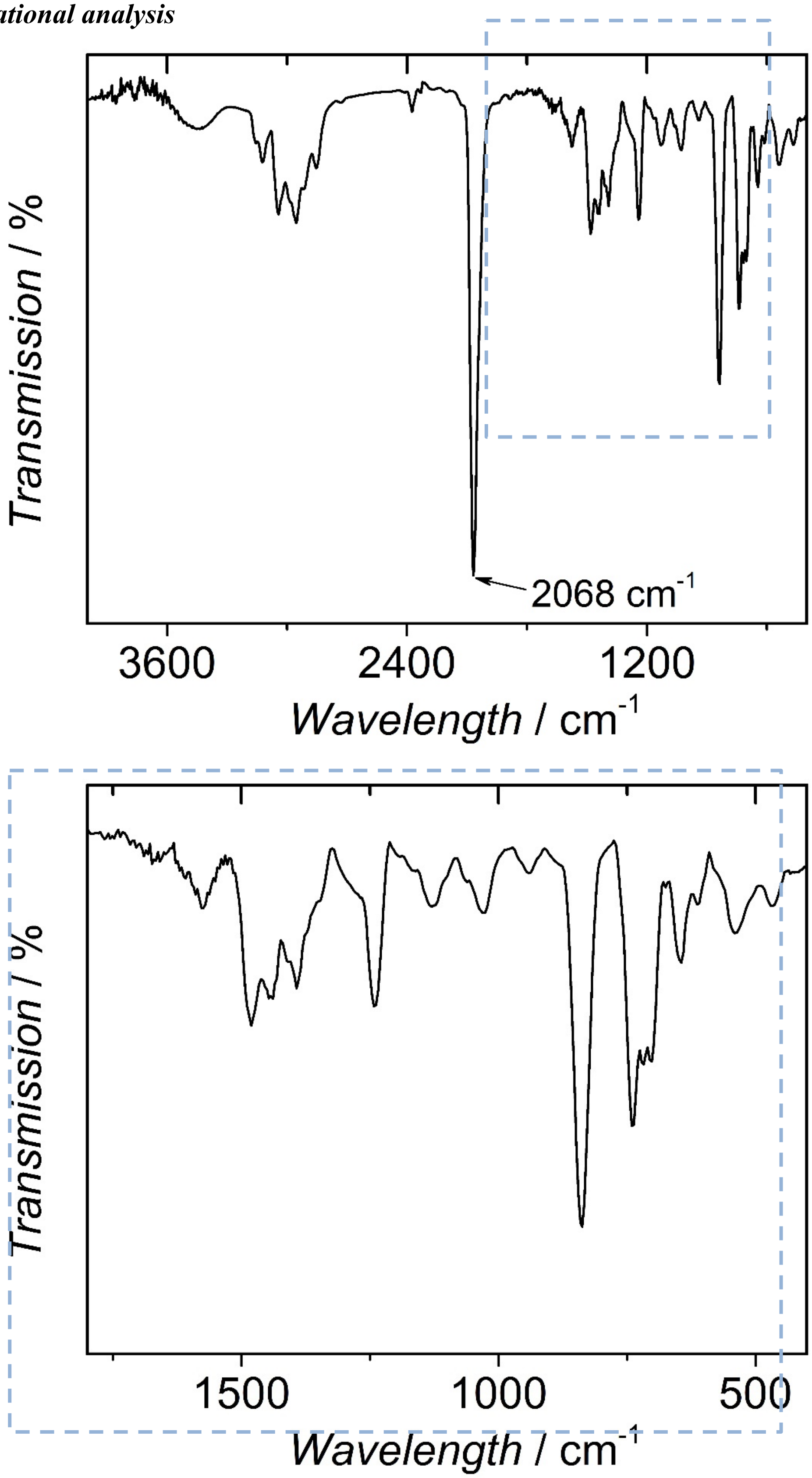

Figure S4. IR vibrational spectrum of **2** measured as KBr pellet.

Electrochemical Analysis of 2.

Cyclic voltammograms of 10 mg of **2** in 5 mL THF (0.1 M NBu_4PF_6) were measured using a glassy carbon working electrode, and platinum wires as counter and *quasi*-reference electrodes. After the initial measurements, ferrocene was added as an internal standard and the voltammograms were corrected by positioning the oxidation potential of ferrocene at 0 V.

The cyclic voltammogram revealed a *quasi*-reversible oxidation $E_{1/2}$ at –0.71 V, and a irreversible reduction $E_{p,c}$ at –2.6 V (*vs.* $FeCp_2^+/FeCp_2$) (Figure S5). According to the Randles-Sevcik analysis, the oxidation of **2** is *quasi*-reversible (Figure S6).

The presence of the anodic wave at $E_{p,a}$ = –1.78 V and the retention of voltammograms over multiple CV cycles hint at chemical reversibility of the reduction event as well. Voltammograms run at mild cathodic potentials (up to –2.4 V) without initiating the cathodic wave at −2.6 V were lacking the anodic wave at –1.78 V, thus confirming that these two waves are related despite being separated by over 800 mV. Overall, the CV analysis indicates that the reduction of **2** at −2.6 V results in concomitant nitrilimine ligand dissociation and the formation of the Fe(I) complex. Subsequently, the oxidation of the Fe(I) complex at −1.78 V leads to nitrilimine (re-) coordination; thereby restoring complex **2**. The CV results corroborate that the Fe(I) complex does not react with Li[Me_3Si–CN_2].

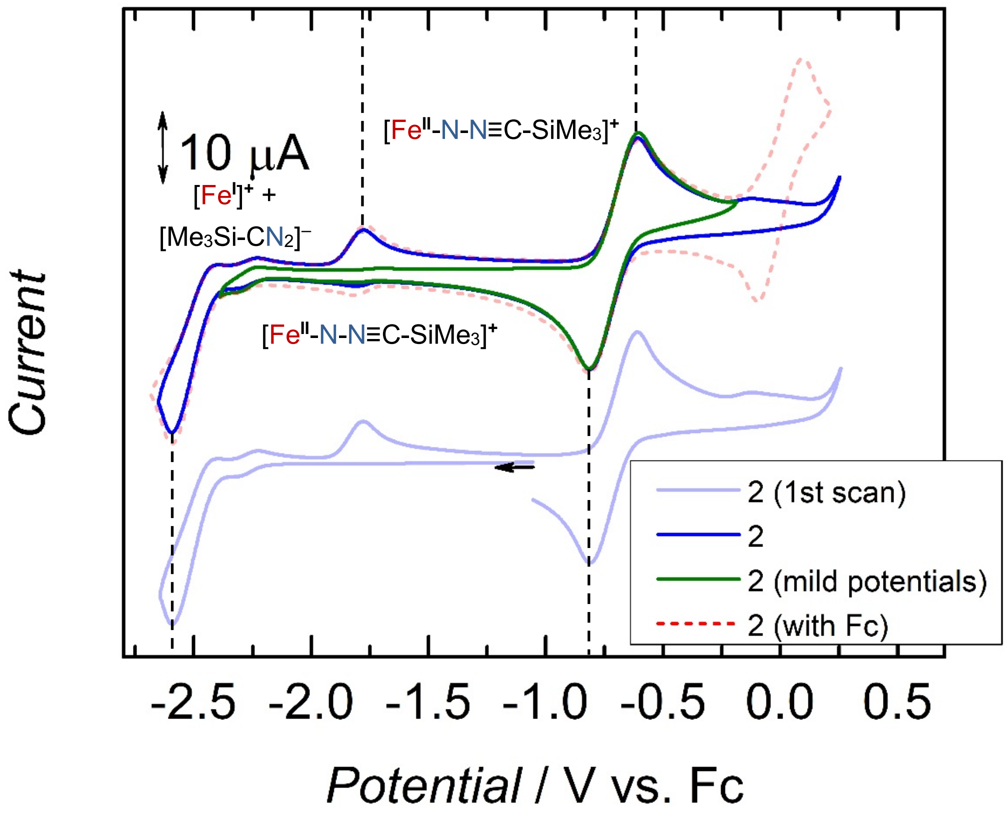

Figure S5. Cyclic voltammograms for **2** with and without ferrocene as an internal standard, measured at 100 mV s^{-1} potential scan rate in 0.1 M NBu_4PF_6 THF solution.

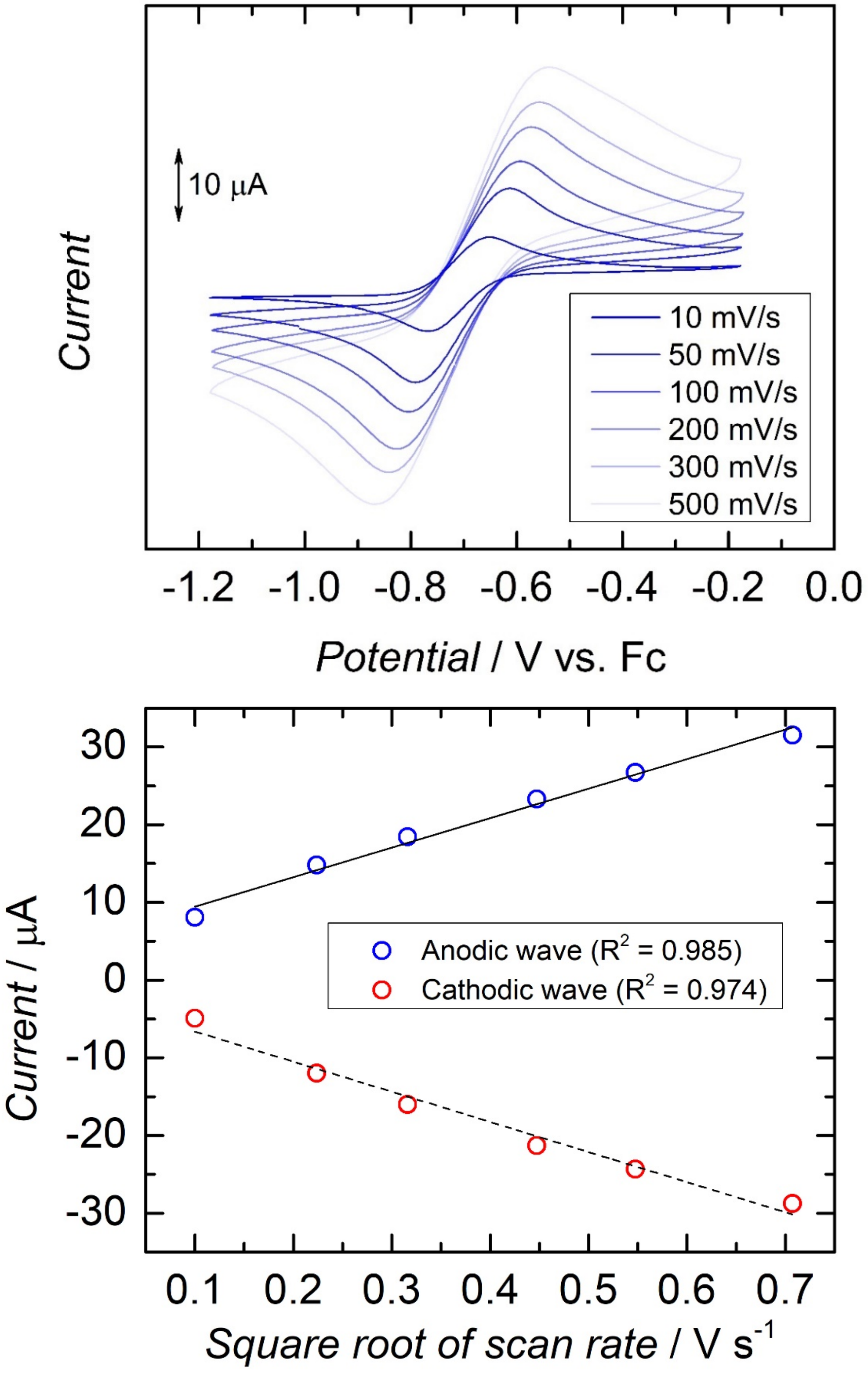

Figure S6. Cyclic voltammograms of the oxidation of **2** at different voltage scan rates (upper panel) and plots of the Randles-Sevcik analysis (lower panel) indicating its *quasi*-reversible character.

Electronic Absorption Spectrum of 2

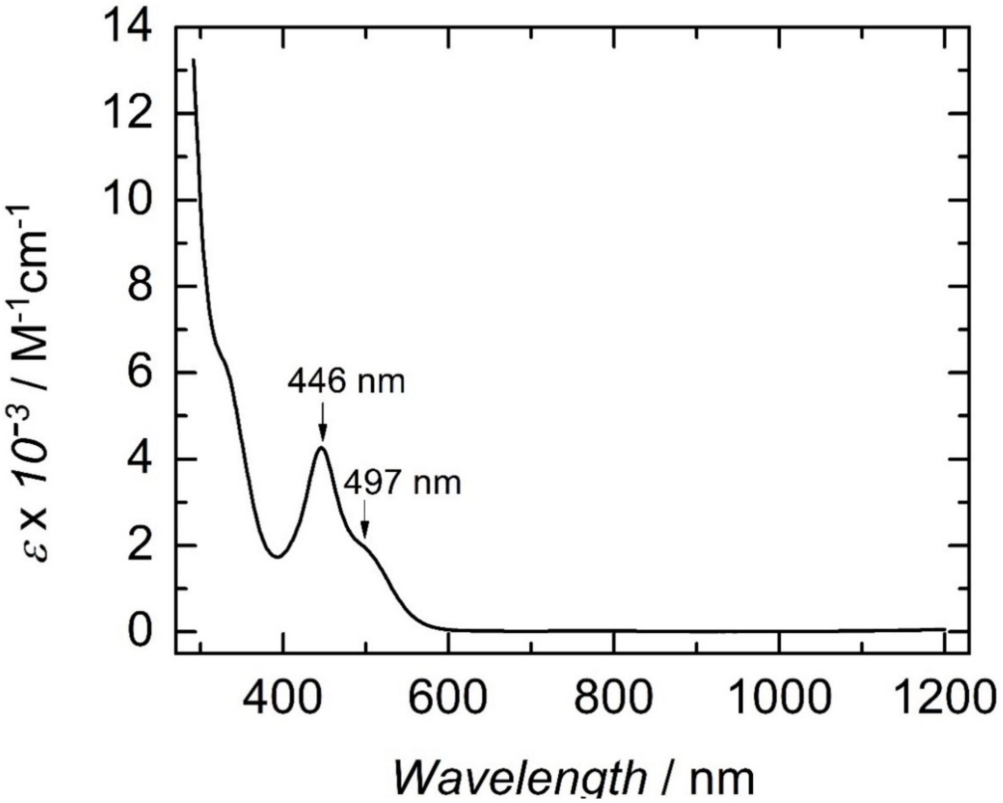

Figure S7. UV-Vis absorption spectrum of **2** in THF.

Reduction of Iron Nitrilimine Complex 2 to Iron Complex 3

***Synthesis of [TIMEN^{mes}Fe](BPh$_4$) (3) by reduction of [TIMEN^{mes}Fe(N$_2$C-SiMe$_3$)](BPh$_4$)* (2)**

KC_8 (2.8 mg, 0.021 mmol, 1.2 equiv.) was added to a cold (−30 °C) orange solution of **2** (20 mg, 0.018 mmol) in *ca.* 2 mL of THF-d_8. Instant darkening of the reaction mixture indicated an immediate reaction. The solution was filtered through a glass-microfiber filter into a J-Young NMR tube. The NMR spectroscopic analysis corroborated quantitative conversion to complex **4**.[5] The additional signal at −0.06 ppm indicates nitrilimine ligand release. Dark red single crystals suitable for XRD analysis were obtained by vapor diffusion of diethyl ether into the THF solution at room temperature.

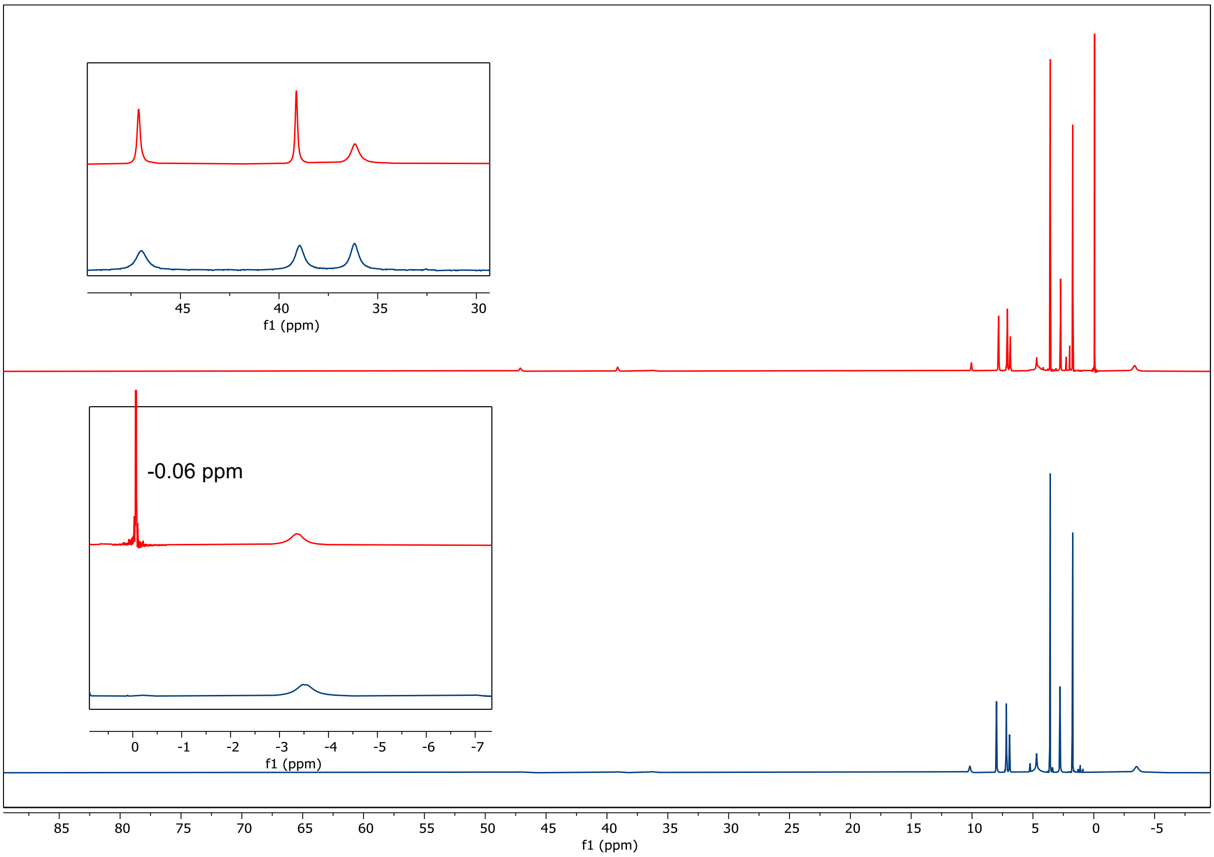

Figure S8. ^{1}H NMR spectra of the mixture of **2** and KC_8 (red), and the independently synthesized complex **3** [TIMEN^{Mes}Fe](BPh$_4$) (blue) in THF-d_8.

Synthesis of [TIMEN^{mes}Fe](BPh$_4$) (3) by reduction of [TIMEN^{mes}FeCl](BPh$_4$) (1)

Sodium amalgam (1 % w/w, 779 mg, 0.339 mmol, 1.2 equiv.) was added to a solution of **1** (300 mg, 0.282 mmol) in 4 mL of THF, and the mixture was vigorously stirred overnight. Afterwards, the mixture was filtered through celite® and the solution was concentrated to 2 mL. Diffusion of diethyl ether into a THF solution at −30 °C resulted in crystallization of the dark red product, which was collected, washed with diethyl ether, and dried under high vacuum to afford 215 mg of the product (74 %).

Elemental Analysis (%): Calculated for $C_{66}H_{71}FeN_7B$: C 77.03, H 6.95, N 9.53. Found: C 76.94, H 7.12, N 9.64%.

^{1}H NMR (400 MHz, THF-*d*$_8$): δ 46.98 (s, broad), 38.96 (s, broad), 36.18 (s, broad), 10.18 (s, 2H), 8.00 (s, 8H, BPh_4^-), 7.19 (s, 8H, BPh_4^-), 6.93 (s, 4H, BPh_4^-), 4.70 (s, broad, 8H), 3.59 (s, 9H), 2.78 (s, 9H), 1.73 (s, 9H), -3.51 (s, broad, 6H) ppm.

^{13}C NMR (68 MHz, THF-*d*$_8$): δ 167.42, 166.69, 165.91, 165.24, 138.88, 138.43, 130.23, 126.89, 122.71, 104.78, 39.32, 23.55 ppm.

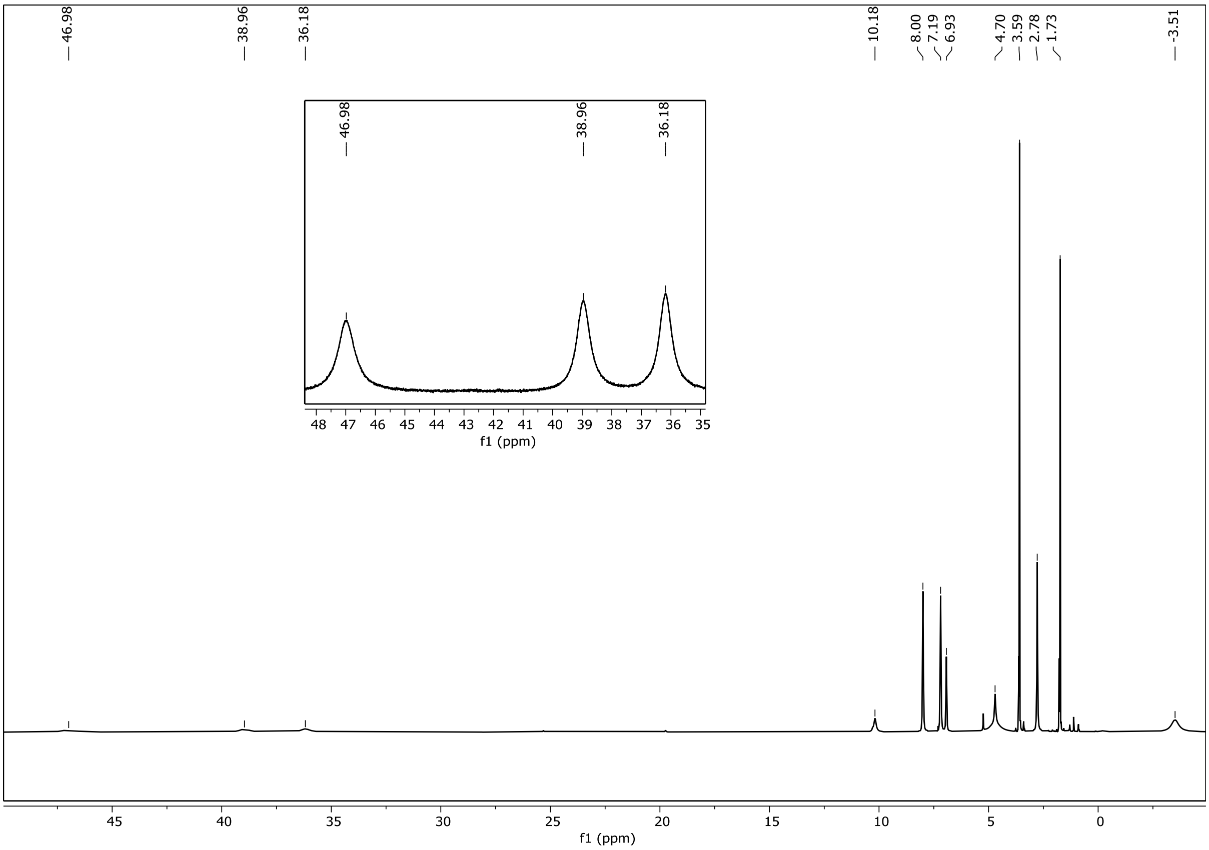

Figure S9. ^{1}H NMR spectrum of **3** in THF-*d*$_8$.

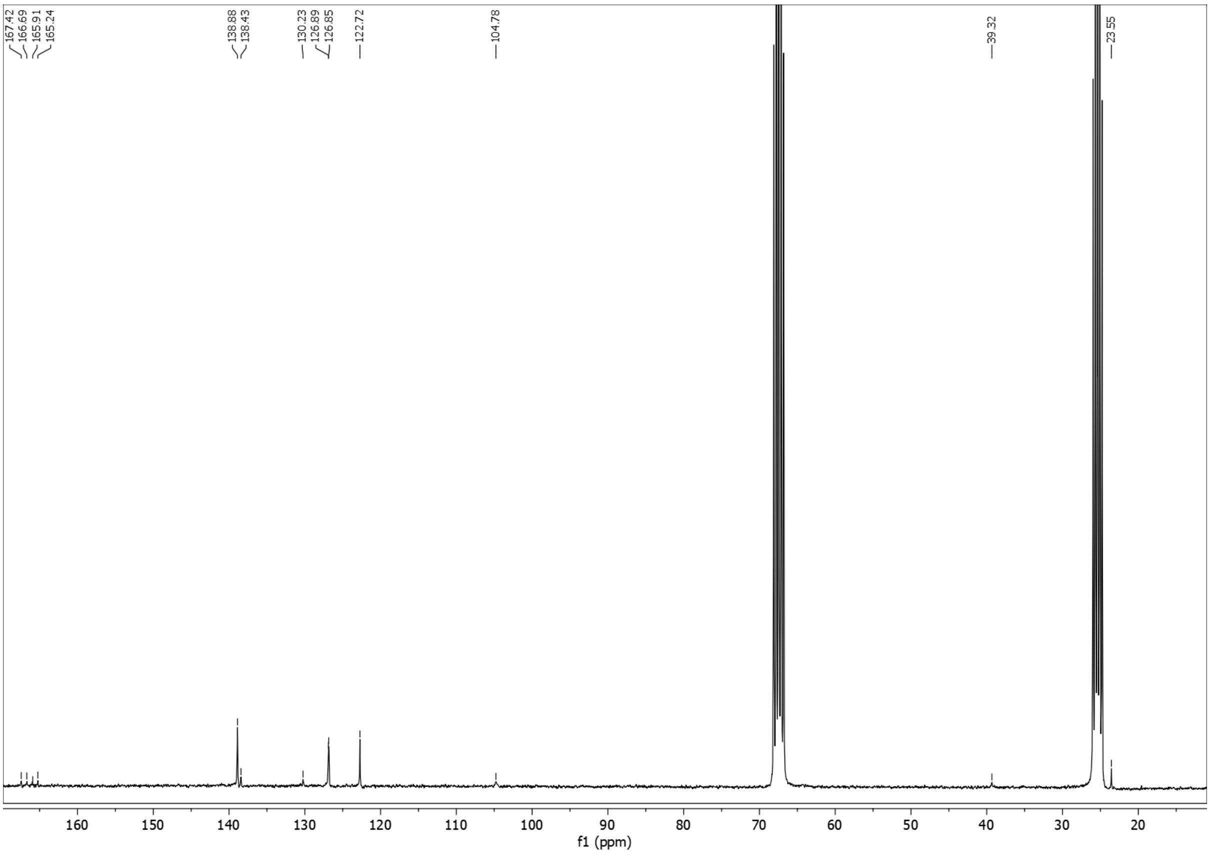

Figure S10. ^{13}C NMR spectrum of **3** in THF-d_8.

N–N Bond Cleavage of Iron Nitrilimine Complex 2 to Iron Nitrido Complex 4

NMR Scale Reaction of 2 with NBu_4F

In a J-Young NMR tube, **2** (20 mg, 0.0175 mmol) was dissolved in *ca.* 0.5 mL of THF-d_8. It was cooled to −30 °C and NBu_4F (5.5 mg, 0.021 mmol, 1.2 equiv.) suspended in ca. 0.5 mL of THF-d_8 was added. An instant color change from orange to purple was observed. The ^{1}H and ^{13}C NMR spectroscopic analysis confirmed quantitative conversion of **2** and showed the formation of [TIMEN^{mes}FeN](BPh_4), as well as the presence of $NBu_4^+CN^-$ (^{13}C NMR: 166 ppm) and TMSF (^{1}H NMR: 0.02 ppm; ^{13}C NMR: −0.4 ppm; ^{19}F NMR: −152 ppm).[6] The formation of minor amounts of unidentified side products (*), removable by recrystallization from THF/Et_2O (see Figure S13), were also observed.

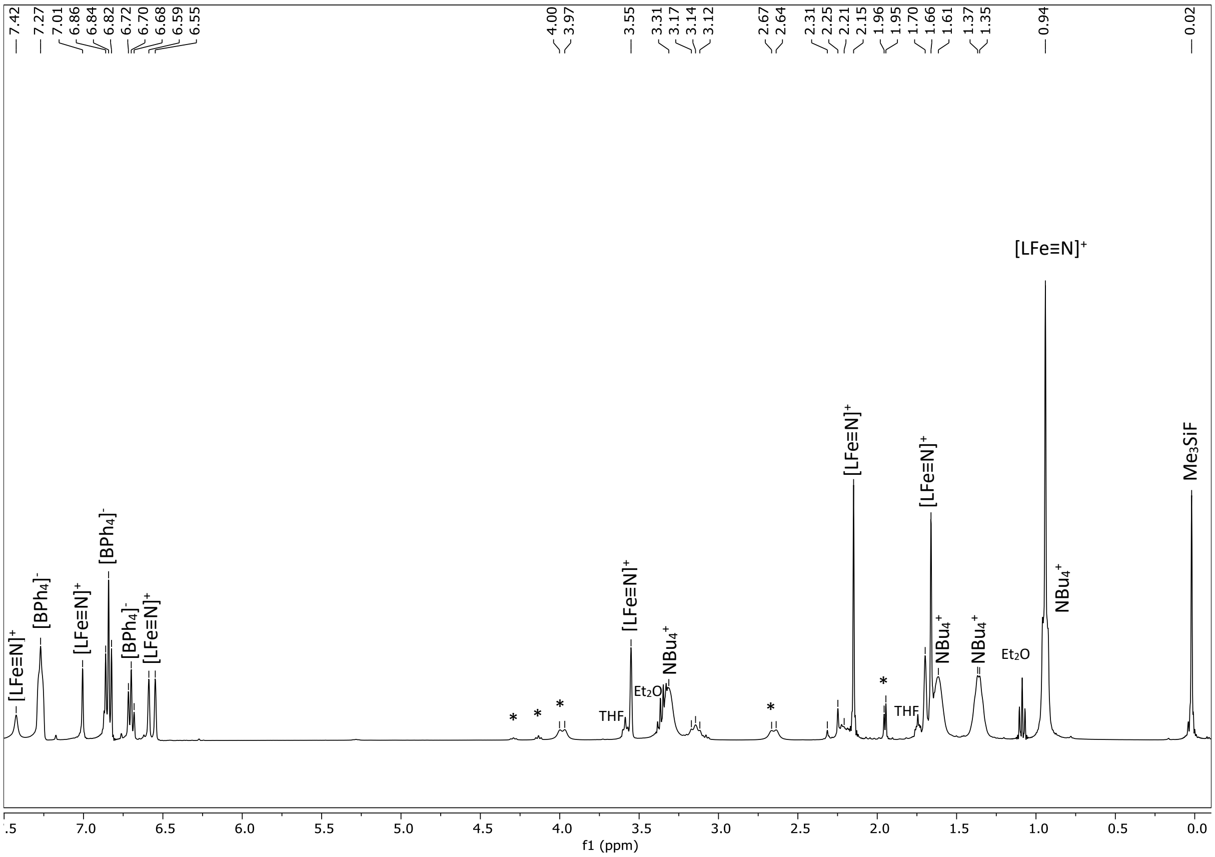

Figure S11. ^{1}H NMR spectrum of the crude reaction mixture in THF-d_8.

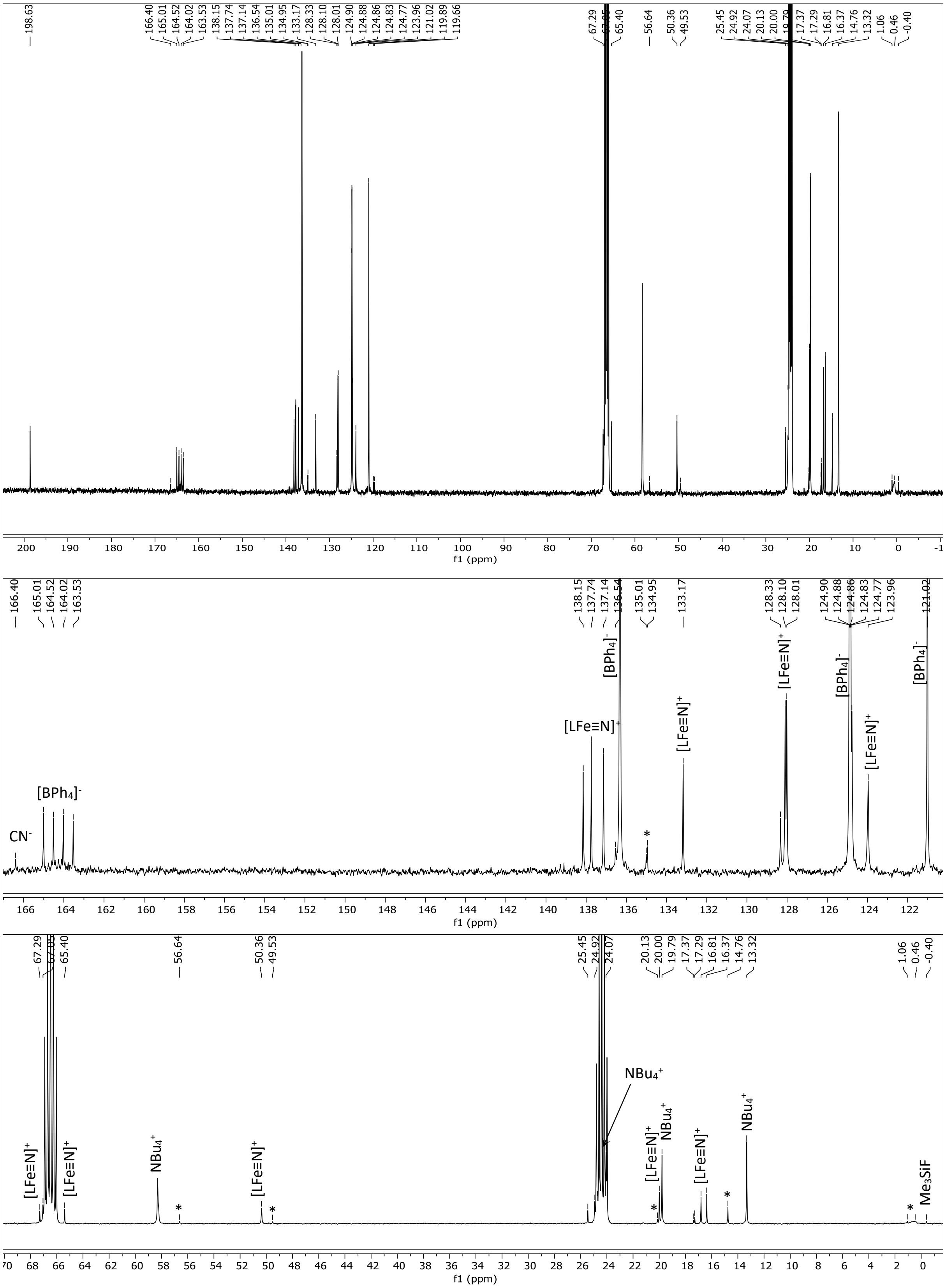

Figure S12. ^{13}C NMR spectra of the crude reaction mixture in THF-d_8.

Synthesis of the iron nitrido complex 4 by reaction of 2 with NBu$_4$F

In a scintillation vial, **2** (50 mg, 0,044 mmol) was dissolved in 4 mL of THF. The solution was cooled to −100 °C and NBu$_4$F (11.5 mg, 0.044 mmol, 1 equiv.) suspended in 4 mL of THF was added. The reaction mixture instantly turned purple. The reaction mixture was filtered after stirring for 1 hour at room temperature. The filtrate was concentrated to *ca.* 1 mL and left at −30 °C overnight, which resulted in the formation of purple needles. The supernatant solution was decanted, diluted with Et$_2$O, and cooled again to −30 °C for 8 hours to obtain a second batch of product. The combined batches were washed with ca. 2 mL of Et$_2$O to give 41 mg of the product (91%).

The ^{1}H NMR spectrum matched the previously published data.[2] Single crystals for XRD analysis were grown by vapor diffusion of Et$_2$O into a solution in THF.

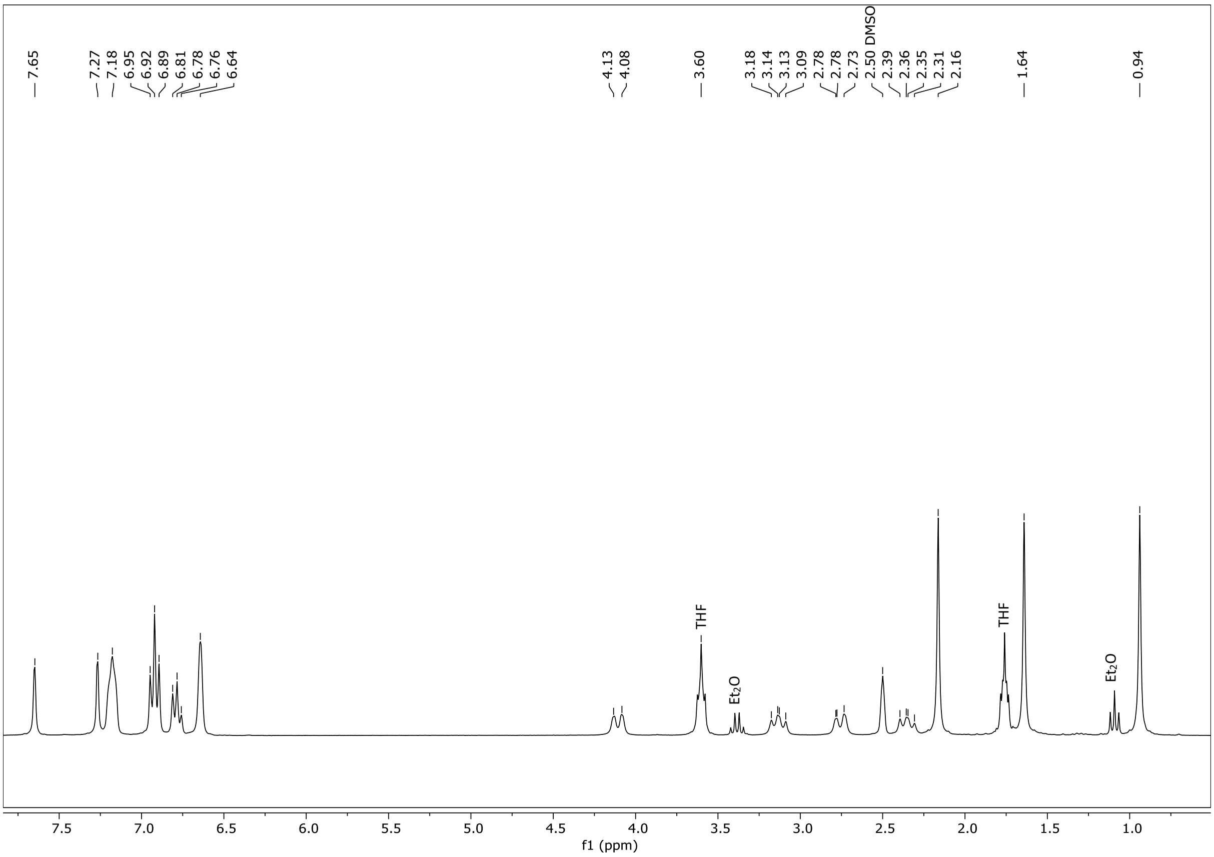

Figure S13. ^{1}H NMR spectrum of the iron nitrido complex **4** in THF-d_8.

Irradiation of 2

Solutions of **2**, either in pyridine-d_5 or THF-d_8, were irradiated in a quartz J-Young NMR tube. A 150 W Xe arc-lamp or two 15 W UV lamps, providing 254 and 365 nm (the sample placed in between the lamps with approx. 1 cm distance to the light sources), were used to irradiate the sample. With both experimental settings, no change in the ^{1}H NMR spectrum could be observed after up to 6 hours of irradiation. Irradiation in benzene-d_6 was not performed due to very low solubility of **2**.

XRD Analyses

X-ray Crystal Structure Determination Details:

Orange plate-shaped crystals of [TIMEN^{mes}Fe(N_2CSiMe_3)](BPh_4)·$(THF)_{3.5}$ were grown by pentane vapor diffusion into a THF solution of **2** at –30 °C. Dark-red needle shaped crystals of [TIMEN^{mes}Fe](BPh_4)·$(THF)_{2.5}$·$(Et_2O)_{0.5}$ were grown by diethyl ether vapor diffusion into a THF solution of **3**. Purple plate-shaped crystals of [(TIMEN^{mes})FeN]-(BPh_4)·(Et_2O) were grown by vapor diffusion of diethyl ether into THF solution of **4** at ambient temperature. Suitable single crystals were embedded in protective perfluoropolyalkylether oil and transferred to the cold nitrogen gas stream of the diffractometer. Intensity data of all three crystals were collected at 100 K using graphite monochromatized MoK_α radiation (λ = 0.71073 Å) on a Bruker Kappa APEX 2 *IμS* Duo diffractometer equipped with QUAZAR focusing Montel optics. Data were corrected for Lorentz and polarization effects, semi-empirical absorption corrections were performed on the basis of multiple scans using SADABS.[7] The structures were solved by direct methods (SHELX XT 2014/5)[8] and refined by full-matrix least-squares procedures on F^2 using SHELXL 2018/3.[9] All non-hydrogen atoms were refined with anisotropic displacement parameters. The hydrogen atoms were placed in positions of optimized geometry, their isotropic displacement parameters were tied to those of the corresponding carrier atoms by a factor of either 1.2 or 1.5.

The compound **2** crystallized with a total of 3.5 molecules of tetrahydrofuran (THF) with one disordered 0.5 THF being situated on a crystallographic inversion center. Similarity and pseudo-isotropic restraints were applied to the anisotropic displacement parameters of the disordered atoms of the solvent molecules. The compound **3** crystallized with a total of 2 molecules of tetrahydrofuran (THF) and 0.5 molecules of Et_2O (diethyl ether) all of which were disordered. Two alternative orientations were refined for the THF molecules and resulted in site occupancies of 39(2) and 61(2) % for the atoms O100 – C104 and O110 – C114; and of 54.1(4) and 45.9(4) % for the atoms O200 – C204 and O210 – C214. The 0.5 Et_2O molecule was situated on a crystallographic inversion center and one carbon atom was disordered over two alternative sites (refined occupancy of 54(2) and 46(2) % for the atoms C301 and C311, respectively. Similarity restraints were applied to the anisotropic displacement parameters of the disordered atoms of the THF solvent molecules and for the atoms C16 – C24. Pseudo-isotropic restraints were applied to the anisotropic displacement parameters of the atoms C22, C40, C41, and C302. The structure determination of **4** suffered from severe disorder affecting all structure parts of the complex salt and the included solvent. The TIMEN^{mes} ligand was completely disordered around the central Fe-N unit. Two alternative orientations were refined and resulted in site occupancies of 68.0(2) and 32.0(2) % for the two orientations of all TIMEN^{mes} ligand atoms with the exception of the anchor nitrogen atom N1. In the BPh_4 anion one of the phenyl groups showed rotational disorder. Three alternative orientations were refined and resulted in site occupancies of 33.5(3), 32.0(2), and 34.7(3) % for the atoms C61 – C66, C61A – C66A, and C61B – C66B, respectively. The compound **4** crystallized with one molecule of Et_2O (diethyl ether) which was disordered. Three alternative orientations were refined and resulted in site occupancies of 35.5(6), 32.3(6), and 32.1(6) % for the atoms C101 – C104, C111 – C114, C121 – C124, respectively. A general RIGU restraint was applied. Additional similarity restraints were applied to the anisotropic displacement parameters of the atoms of the disordered phenyl group and to the atoms Fe1 and N8. Pseudo-isotropic restraints were applied to the anisotropic displacement parameters of some of the disordered atoms.

Table S1. Crystallographic data, data collection, and refinement details.

	**2** · 3.5·THF	**3** · 2·THF · 0.5·Et_2O	**4** · Et_2O
**CCDC**	**1946912**	**1946913**	**1946914**
Empirical formula	$C_{84}H_{108}BFeN_9O_{3.5}Si$	$C_{76}H_{92}BFeN_7O_{2.5}$	$C_{70}H_{81}BFeN_8O$
Mol. Weight	1394.55	1210.24	1117.10
Crystal size [mm]	0.19×0.10×0.04	0.26×0.12×0.07	0.19×0.11×0.04
Temperature [K]	100	100	100
Crystal system	*monoclinic*	*triclinic*	*triclinic*
Space group	$P2_1/c$ (Nr. 14)	$P\bar{1}$ (Nr. 2)	$P\bar{1}$ (Nr. 2)
a[Å]	23.556(2)	11.1777(5)	12.4461(7)
b[Å]	11.7371(6)	17.4060(7)	12.6191(7)
c[Å]	28.122(2)	17.5618(9)	22.611(2)
α[°]	90	85.574(2)	80.049(2)
β[°]	92.701(2)	79.753(2)	84.731(2)
γ[°]	90	77.508(2)	61.148(2)
V[Å^3]	7766.5(7)	3280.0(3)	3063.5(3)
Z	2	2	2
ρ [g/cm^3] (calc.)	0.596	1.225	1.211
μ [mm^{-1}]	0.264	0.283	0.296
F (000)	2992	1296	1192
Abs. corr.	SADABS	SADABS	SADABS
T_{min} ; T_{max}	0.695; 0.746	0.631; 0.746	0.705; 0.746
2Θ-range [°]	$4.0 \leq 2\Theta \leq 52.1$	$4.0 \leq 2\Theta \leq 52.8$	$4.0 \leq 2\Theta \leq 54.2$
Coll. Refl.	112690	106768	92797
Indep. Refl.	15306	13399	13494
Obs. Ref. $F_o \geq 4.0\sigma(F)$	11253	10142	9985
No. ref. param.	931	902	1355
wR_2 (all data)	0.1761	0.1800	0.1164
R_1 ($F_o \geq 4.0\sigma(F)$)	0.0690	0.0657	0.0485
GooF F^2	1.056	1.077	1.005
Max.; min. res. Elect.	0.625; −0.702	2.200; −0.809	0.998; −0.524

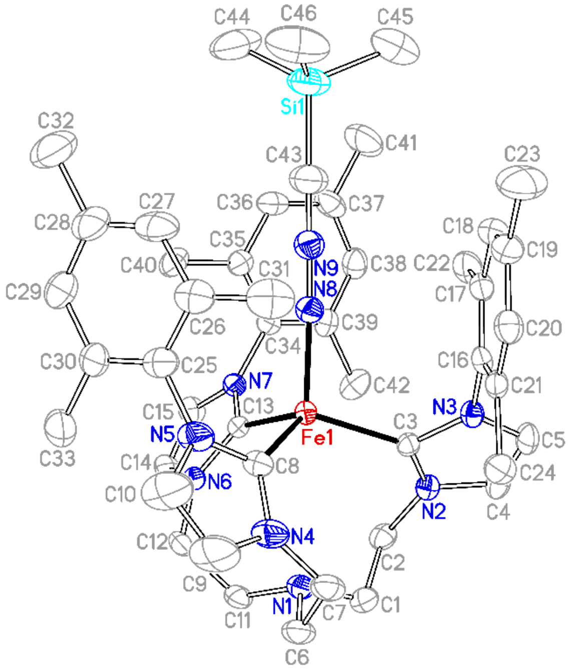

Figure S14. Thermal ellipsoid plot of the complex cation [(TIMEN^{mes})Fe(N_2CSiMe_3)]$^+$ in crystals of [TIMEN^{mes}Fe(N_2CSiMe_3)](BPh_4)·3.5THF (**2**·3.5THF) with labeling scheme (hydrogen atoms and non-coordinated BPh_4^- anion are omitted for clarity).

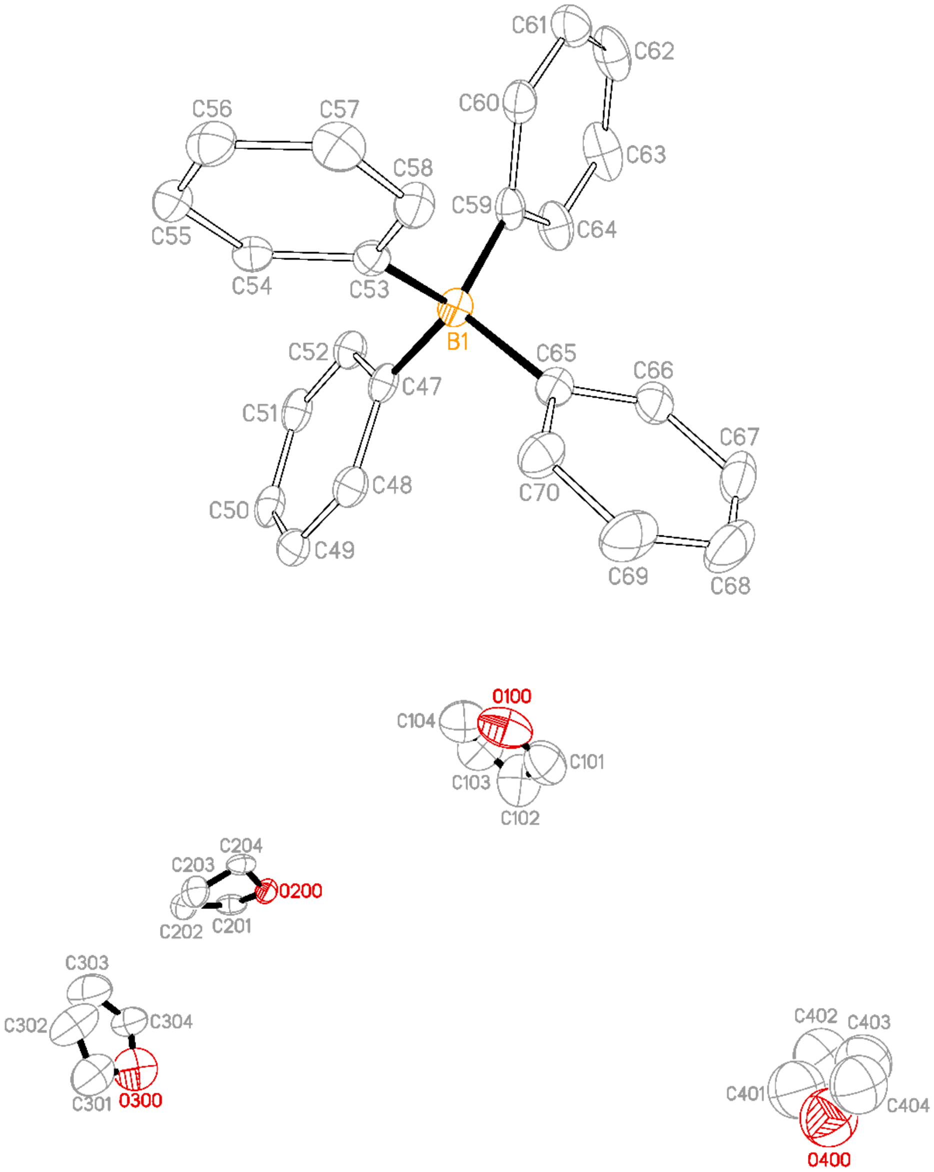

Figure S15. Thermal ellipsoid plot of the BPh_4^- anion and solvent molecules of $[(TIMEN^{mes})Fe(N_2CSiMe_3)]$-$(BPh_4)\cdot 3.5THF$ (**2**·3.5THF) with labeling scheme (hydrogen atoms omitted for clarity).

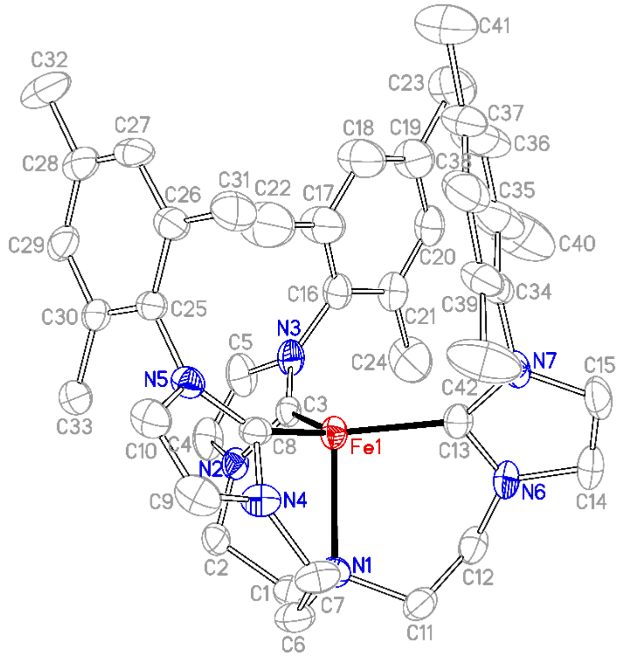

Figure S16. Thermal ellipsoid plot of the complex cation [(TIMEN^{mes})Fe]$^{+}$ in crystals of [(TIMEN^{mes})Fe]-(BPh_4) · 2THF · 0.5Et_2O (**3**·2THF ·0.5Et_2O) with labeling scheme (hydrogen atoms and non-coordinated BPh_4^- anion are omitted for clarity).

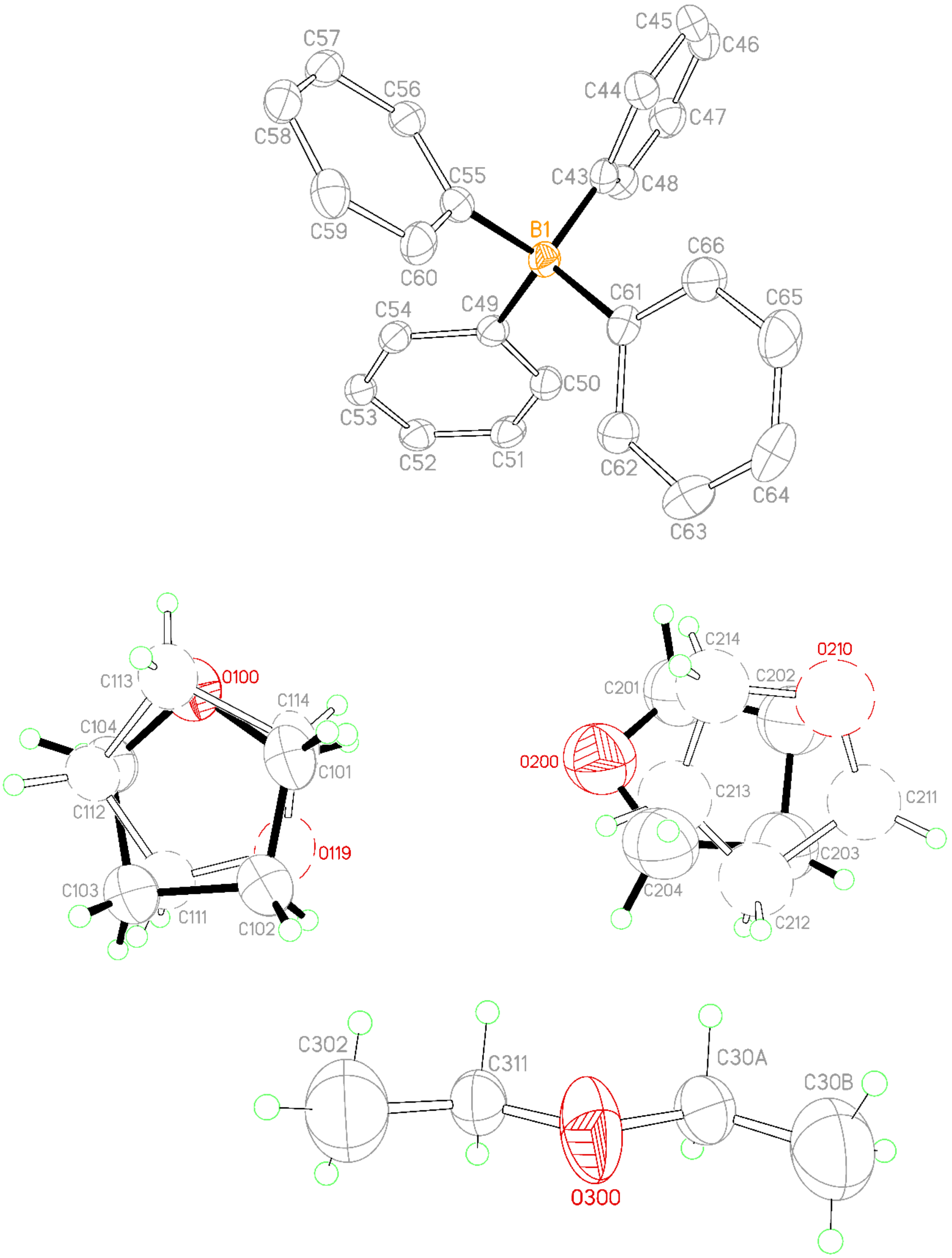

Figure S17. Thermal ellipsoid plot of the BPh_4^- anion and solvent molecules of [(TIMEN^{mes})Fe]-(BPh_4) · 2THF · 0.5Et_2O (**3**·2THF ·0.5Et_2O) with labeling scheme and presentation of the disorder of the tetrahydrofuran solvent molecules.

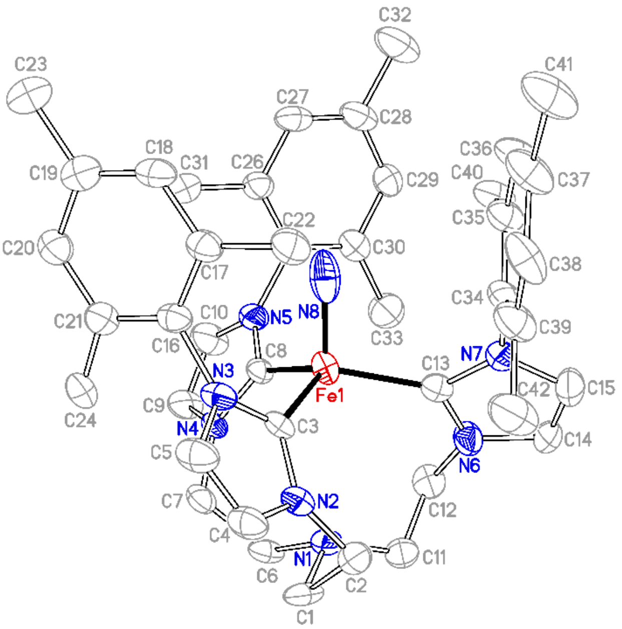

Figure S18. Thermal ellipsoid plot of the complex cation [(TIMEN^{mes})FeN]$^{+}$ in crystals of [(TIMEN^{mes})FeN]-(BPh$_4$)· Et$_2$O (**4**·Et$_2$O) with labeling scheme (disorder of the ligand and hydrogen atoms and non-coordinated BPh$_4^{-}$ anion are omitted for clarity).

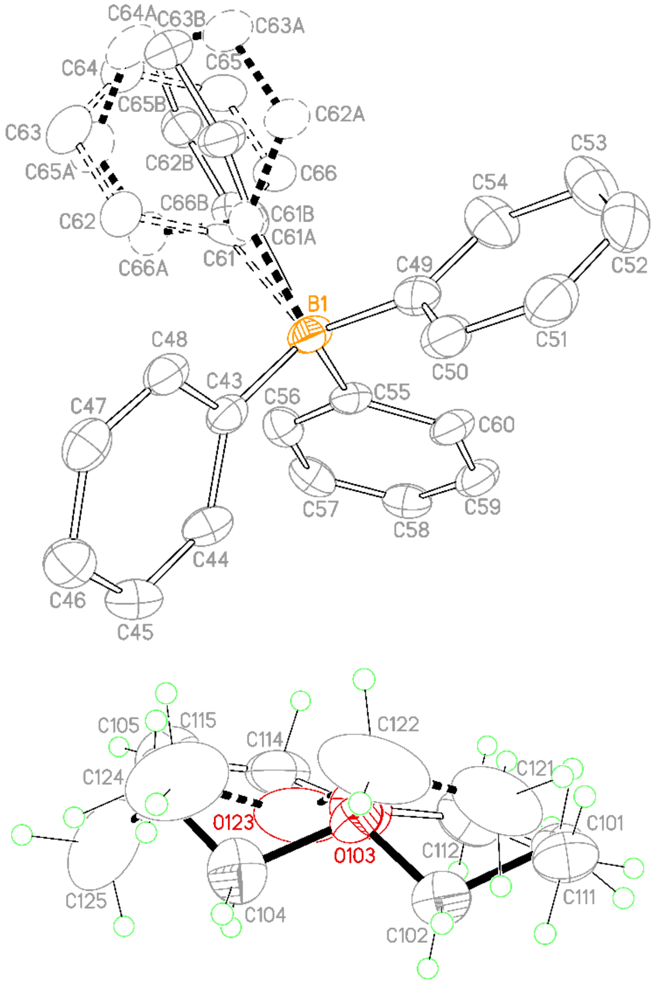

Figure S19. Thermal ellipsoid plot highlighting the observed disorder of the BPh_4^- anion and the solvent molecules of [/TIMEN^{mes})FeN](BPh_4) Et_2O (**4**·Et_2O) with labeling scheme.

Computational Details

All calculations were performed with ORCA v. 4.0.1.[10] The geometric parameters of **2** were optimized using the BP86 functional,[11] with dispersion correction D3(BJ)[12] and the ZORA-def2-SVP basis set.[13] The ZORA-def2-TZVP basis set was used for iron. Scalar relativistic effects were modeled with the Zeroth Order Regular Approximation (ZORA) as implemented in ORCA.[14] The resolution of identity approximation and the related relativistically recontracted auxiliary basis set ("*SARC/J*")[15] were used to speed up the calculations. Tighter than default scf ("*tightscf*") and optimization criteria ("*tightopt*") were chosen in conjunction with finer than default grid values ("*grid5*"; "*finalgrid6*"). The optimized geometric parameters were verified as true minima by the absence of negative eigenvalues in the harmonic vibrational frequency analysis. The calculated N–N bond stretching frequency was scaled by 0.981 as suggested in the literature.[16] Various electronic states (including broken symmetry states) were investigated. The energies of the optimized structures did not allow for the unambiguous assignment of the spin states (Table S2). Compared to the solid-state molecular structure, obtained from an XRD study, only the quintet state led to satisfying structural parameters. Single point calculations with the ZORA-def2-TZVPP basis set on all atoms were subsequently performed. The calculation of the ^{57}Fe Mößbauer parameters was performed with the core properties basis set CP(PPP)[17] for iron and the DKH-def2-TZVPP basis set for all other atoms, the second order Douglas-Kroll-Hess method,[18] and even tighter scf ("*verytightscf*") and grid setting for iron (*"SpecialGridIntAcc 7"*) and the other atoms ("*grid6*", "*nofinalgrid*"). The ^{57}Fe Mößbauer isomer shift was calibrated using the extended ("whole") calibration parameters according to Neese, which includes previously reported calibration sets.[19]

For the CASSCF calculations, the structural parameters of the solid state structure were used. The mesityl groups were truncated by methyl groups and the positions of all hydrogen atoms were geometry optimized (BP86/def2-SVP). For the subsequent CASSCF calculations, the ZORA-def2-TZVPP basis set, the RIJCOSX approximation[20] and the related relativistically recontracted auxiliary basis set ("*SARC/J*") were used to speed up the calculations. Various active spaces were investigated, but an active space of 10 electrons in 9 orbitals (5 d-orbitals, 2 π-orbitals of nitrilimine, 2 π*-orbitals of nitrilimine) proved most suitable. For the calculation of the energy of the different electronic states, state averaged calculations (singlet, triplet, quintet; each with 10 roots) were performed and correction by second order perturbation theory (NEVPT2) was applied.[21] For the plot of the molecular orbitals, only the first two quintet roots, which correspond to a d–d transition, were included.

Table S2. Different electronic energies found by the CASSCF/NEVPT2 and DFT calculations. The given values correspond to the vertical transitions found by CASSCF and the adiabatic energies found by DFT.

**Singlet**	**Triplet**	**Quintet**
2.8 eV (64.6 kcal mol^{-1})	1.7 eV (39.2 kcal mol^{-1})	0 eV (39.2 kcal mol^{-1})
+4.5 kcal mol^{-1}	−0.3 kcal mol^{-1}	0 kcal mol^{-1}

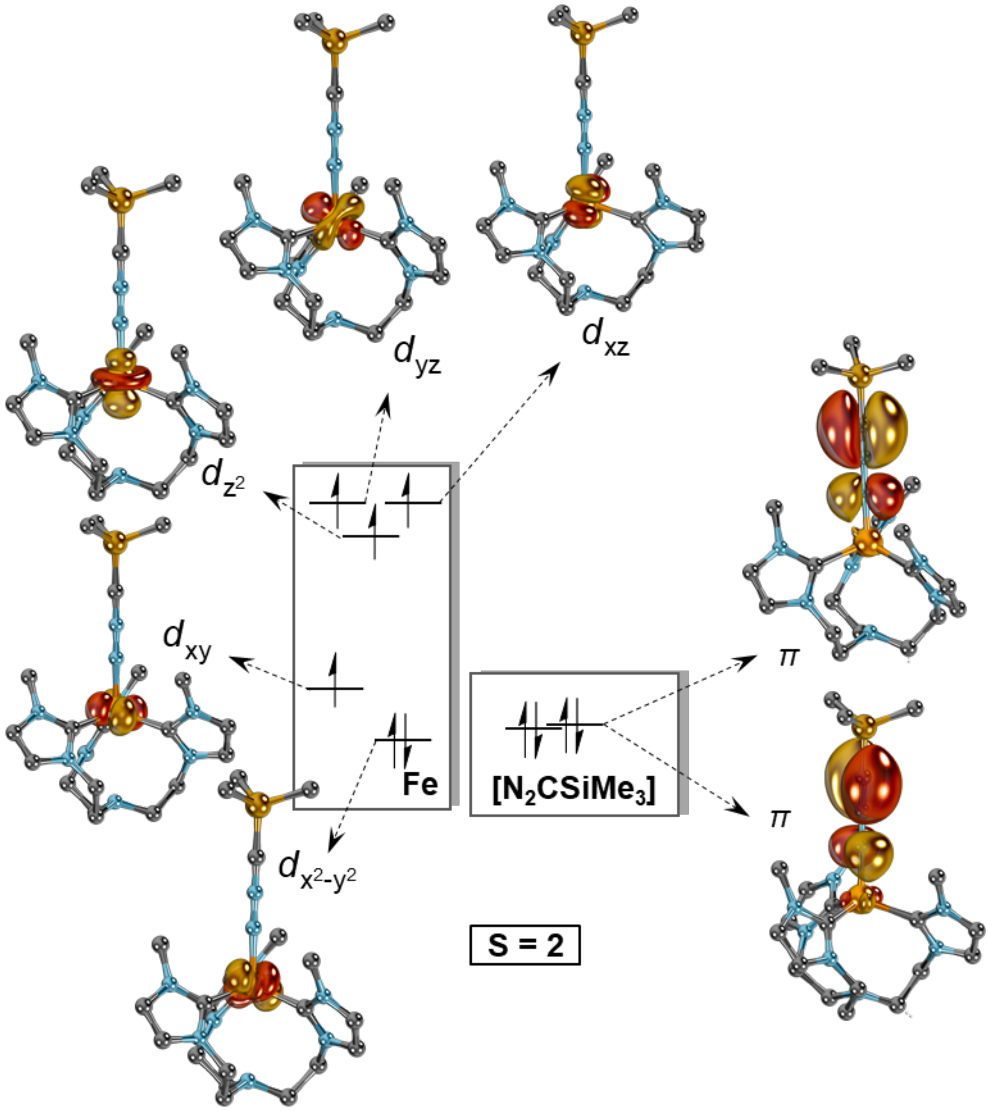

Figure S20. Electronic structure of truncated cation of **2** as predicted from CASSCF(10,9) calculations.

XYZ Coordinates

No truncation

NImag = 0

26	-0.330607000	-1.094833000	0.183364000
7	-1.409646000	-4.578020000	0.412279000
7	-3.231690000	-1.985757000	0.904018000
7	-2.511530000	-0.339677000	2.108042000
7	-0.313237000	-3.068260000	-2.180170000
7	-1.038733000	-1.115408000	-2.765645000
6	-2.854186000	-4.416684000	0.373718000
1	-3.271225000	-4.605794000	1.381345000
1	-3.314306000	-5.173439000	-0.299371000
6	-3.338651000	-3.036756000	-0.105084000
1	-2.751252000	-2.715088000	-0.979835000
1	-4.398694000	-3.100987000	-0.408946000
6	-2.146654000	-1.155788000	1.061215000
6	-4.235067000	-1.698170000	1.825387000
1	-5.181762000	-2.238778000	1.853840000
6	-3.780413000	-0.648399000	2.584120000
1	-4.245186000	-0.093923000	3.399478000
6	-0.776512000	-5.109992000	-0.784910000
1	-1.555756000	-5.350355000	-1.534154000
1	-0.260929000	-6.068380000	-0.556279000
6	0.261161000	-4.183728000	-1.438838000
1	0.922543000	-3.759563000	-0.667285000
1	0.882660000	-4.769877000	-2.140054000
6	-0.525221000	-1.797493000	-1.678697000
6	-0.701710000	-3.170865000	-3.516530000
1	-0.607541000	-4.095597000	-4.086590000
6	-1.147251000	-1.931246000	-3.889447000
1	-1.511723000	-1.553161000	-4.844636000
6	-1.724492000	0.772767000	2.583625000
6	-1.890129000	2.019771000	1.940562000
6	-1.137700000	3.105888000	2.417552000
1	-1.254948000	4.078084000	1.925157000
6	-0.239945000	2.977145000	3.493833000
6	-0.098230000	1.713247000	4.095381000
1	0.602913000	1.590396000	4.931721000
6	-0.831436000	0.593311000	3.660237000
6	-2.788923000	2.163084000	0.741980000
1	-2.356344000	1.620951000	-0.118305000
1	-2.895710000	3.220145000	0.451948000
1	-3.797335000	1.747877000	0.919644000
6	0.530763000	4.168571000	4.009256000

1	0.059773000	4.577883000	4.923141000
1	0.565096000	4.981623000	3.265103000
1	1.568109000	3.897715000	4.273972000
6	-0.608183000	-0.754519000	4.298981000
1	-0.281257000	-0.646135000	5.346462000
1	0.186452000	-1.302390000	3.758444000
1	-1.515861000	-1.381080000	4.286326000
6	-1.349891000	0.290036000	-2.796255000
6	-0.298731000	1.199291000	-3.042891000
6	-0.619589000	2.565599000	-3.119836000
6	-1.937834000	3.033304000	-2.967863000
6	-2.954447000	2.091180000	-2.721778000
1	-3.989043000	2.436135000	-2.591314000
6	-2.685855000	0.713135000	-2.629019000
6	1.122431000	0.720436000	-3.175238000
1	1.227901000	-0.048236000	-3.962777000
1	1.464018000	0.264132000	-2.229370000
1	1.800792000	1.555060000	-3.411578000
6	-3.786945000	-0.261568000	-2.293654000
1	-3.766834000	-0.493533000	-1.212425000
1	-3.684090000	-1.215698000	-2.838458000
1	-4.777123000	0.163076000	-2.526979000
1	0.188989000	3.281415000	-3.306251000
6	-2.260776000	4.504334000	-3.080472000
1	-2.797338000	4.722699000	-4.023002000
1	-2.912961000	4.839849000	-2.254009000
1	-1.347284000	5.121975000	-3.069148000
6	3.320974000	-2.502057000	-1.852166000
7	1.009527000	-3.206071000	1.968046000
7	2.461206000	-2.002379000	0.901280000
6	-0.807364000	-4.931310000	1.687905000
1	0.005633000	-5.665046000	1.520720000
1	-1.550793000	-5.437371000	2.340466000
6	-0.242697000	-3.745939000	2.488435000
1	-0.978481000	-2.925935000	2.497865000
1	-0.067335000	-4.056254000	3.534524000
6	1.103887000	-2.160424000	1.073426000
6	2.268619000	-3.692878000	2.321088000
1	2.405591000	-4.513396000	3.026096000
6	3.186125000	-2.926164000	1.651333000
1	4.276066000	-2.938796000	1.655453000
6	3.067300000	-0.934870000	0.147068000
6	3.201401000	0.324513000	0.773370000
6	3.832664000	1.350324000	0.048197000

6	4.333433000	1.148558000	-1.250808000
6	4.174488000	-0.121483000	-1.836557000
1	4.548815000	-0.296401000	-2.854174000
6	3.539838000	-1.179341000	-1.160722000
6	2.660851000	0.571272000	2.157877000
1	2.988990000	-0.203583000	2.874285000
1	1.555873000	0.561252000	2.146491000
1	2.984272000	1.553419000	2.537055000
1	2.323766000	-2.515455000	-2.331105000
1	3.366189000	-3.355645000	-1.155171000
1	4.071818000	-2.664013000	-2.643083000
1	3.939609000	2.333282000	0.520983000
6	5.034961000	2.257705000	-1.997644000
1	4.963605000	3.217362000	-1.459508000
1	6.108748000	2.029414000	-2.132490000
1	4.607683000	2.397510000	-3.007387000
7	0.119502000	0.664425000	-0.004952000
7	0.524122000	1.797071000	-0.106729000
6	0.924748000	2.935772000	-0.217080000
14	1.479829000	4.671100000	-0.354836000
6	0.018255000	5.833425000	-0.027272000
1	-0.865219000	5.531280000	-0.615619000
1	-0.263643000	5.842767000	1.040006000
1	0.286378000	6.867360000	-0.312925000
6	2.154843000	4.988168000	-2.097694000
1	2.854780000	4.192547000	-2.404717000
1	1.344855000	5.045233000	-2.845322000
1	2.698034000	5.951029000	-2.117610000
6	2.852091000	5.000546000	0.911657000
1	3.038817000	6.087449000	0.991287000
1	2.573749000	4.625145000	1.911082000
1	3.800211000	4.519245000	0.614142000

Truncated Model System

26	16.182800000	2.539478000	18.202346000
14	22.233025000	2.338438000	18.963448000
7	19.272626000	2.445093000	18.615925000
7	18.075477000	2.466497000	18.467059000
7	16.044496000	-0.244891000	19.589887000
7	14.534475000	-0.149803000	18.086135000
7	16.546264000	2.439896000	15.144514000
7	14.823222000	3.617632000	15.582843000
7	16.011113000	4.955015000	20.132417000
7	14.288311000	3.728152000	20.433738000

7	12.917753000	2.553208000	17.790432000
6	23.072622000	3.187829000	17.532746000
6	22.707213000	3.172611000	20.546079000
6	22.859640000	0.615877000	19.109414000
6	20.475460000	2.390686000	18.748487000
6	15.349512000	-1.443015000	19.644835000
6	14.403888000	-1.374672000	18.704779000
6	15.545456000	0.584938000	18.623671000
6	13.592615000	0.303439000	17.052879000
6	12.491646000	1.157917000	17.643281000
6	15.968404000	2.752547000	13.933825000
6	14.888091000	3.490720000	14.206719000
6	15.827773000	2.946475000	16.192377000
6	13.726740000	4.301708000	16.255844000
6	12.593722000	3.350295000	16.594852000
6	15.325339000	5.483286000	21.211668000
6	14.256115000	4.711656000	21.409153000
6	15.381951000	3.844277000	19.635042000
6	13.180596000	2.786133000	20.226337000
6	12.355545000	3.145947000	19.005738000
1	22.854057000	2.687603000	16.571997000
1	22.760798000	4.243902000	17.441920000
1	24.168813000	3.172016000	17.678018000
1	22.409993000	4.236565000	20.578594000
1	22.250324000	2.670465000	21.418207000
1	23.805004000	3.130192000	20.674995000
1	22.424855000	0.095250000	19.981891000
1	22.656967000	-0.001727000	18.215614000
1	23.956158000	0.642298000	19.252184000
1	15.609614000	-2.240673000	20.341167000
1	13.644765000	-2.100256000	18.410343000
1	14.162186000	0.868654000	16.294778000
1	13.179401000	-0.598178000	16.568972000
1	12.226528000	0.741895000	18.632183000
1	11.568137000	1.078040000	17.026912000
1	16.397217000	2.443309000	12.980183000
1	14.163245000	3.962155000	13.542120000
1	14.123826000	4.767946000	17.174896000
1	13.375868000	5.111278000	15.591911000
1	12.435249000	2.677665000	15.732392000
1	11.643334000	3.912747000	16.725305000
1	15.679351000	6.360423000	21.754551000
1	13.458239000	4.775590000	22.149633000
1	13.600519000	1.769685000	20.129772000

1	12.563352000	2.808225000	21.140012000
1	12.347644000	4.246523000	18.906578000
1	11.293732000	2.841930000	19.157584000
6	17.782483000	1.658314000	15.292137000
1	17.792749000	1.216229000	16.300224000
1	17.811143000	0.865134000	14.528008000
1	18.667649000	2.307906000	15.184044000
6	17.178306000	0.092429000	20.462579000
1	16.959240000	-0.226106000	21.494687000
1	18.100260000	-0.402352000	20.112186000
1	17.329640000	1.181154000	20.421207000
6	17.256839000	5.514556000	19.588376000
1	17.997538000	5.640317000	20.394841000
1	17.650204000	4.800665000	18.847961000
1	17.061530000	6.490083000	19.111819000

References

[1] H. Sun, S. G. DiMagno, *J. Am. Chem. Soc.* **2005**, *127*, 2050.

[2] C. Vogel, F. W. Heinemann, J. Sutter, C. Anthon, K. Meyer, *Angew. Chem. Int. Ed.* **2008**, *47*, 2681.

[3] W. J. Evans, E. Montalvo, T. M. Champagne, J. W. Ziller, A. G. DiPasquale, A. L. Rheingold, *J. Am. Chem. Soc.* **2008**, *130*, 16.

[4] G. A. Bain, J. F. Berry, *J. Chem. Educ.* **2008**, *85*, 532.

[5] C. S. Vogel, *High- and Low-Valent tris-N-Heterocyclic Carbene Iron Complexes: A Study of Molecular and Electronic Structure* in *Springer Theses*, **2012**.

[6] P. Dohlert, S. Enthaler, *J. Appl. Polym. Sci.* **2015**, *132*, 42814.

[7] Bruker AXS, Inc. *SADABS 2014/5, Bruker AXS area detector scaling and absorption correction.*, 2014.

[8] G. M. Sheldrick, *Acta Cryst.* **2008,** *A64*, 112.

[9] G. A Sheldrick, *Acta Cryst.* **2015,** *C71*, 3.

[10] a) F. Neese, *Wiley Interdiscip. Rev.: Comput. Mol. Sci.* **2018**, *8*, e1327; b) F. Neese, *Wiley Interdiscip. Rev.: Comput. Mol. Sci.* **2012**, *2*, 73.

[11] a) J. P. Perdew, *Phys. Rev. B* **1986**, *33*, 8822; b) A. D. Becke, *Phys. Rev. A* **1988**, *38*, 3098.

[12] a) S. Grimme, S. Ehrlich, L. Goerigk, *J. Comput. Chem.* **2011**, *32*, 1456; b) S. Grimme, J. Antony, S. Ehrlich, H. Krieg, *J. Chem. Phys.* **2010**, *132*, 154104.

[13] F. Weigend, R. Ahlrichs, *Phys. Chem. Chem. Phys.* **2005**, *7*, 3297.

[14] a) E. v. Lenthe, E. J. Baerends, J. G. Snijders, *J. Chem. Phys.* **1993**, *99*, 4597; b) C. van Wüllen, *J. Chem. Phys.* **1998**, *109*, 392.

[15] F. Weigend, *Phys. Chem. Chem. Phys.* **2006**, *8*, 1057.

[16] M. Alecu, J. Zheng, Y. Zhao, D. G. Truhlar, *J. Chem. Theory Comput.* **2010**, *6*, 2872.

[17] F. Neese, *Inorg. Chim. Acta* **2002**, *337*, 181-192.

[18] A. Wolf, M. Reiher, B. A. Hess, *J. Chem. Phys.* **2002**, *117*, 9215.

[19] a) R. Bjornsson, F. Neese, S. DeBeer, *Inorg. Chem.* **2017**, *56*, 1470; b) M. Römelt, S. Ye, F. Neese, *Inorg. Chem.* **2009**, *48*, 784; c) T. V. Harris, R. K. Szilagyi, *Inorg. Chem.* **2011**, *50*, 4811.

[20] a) F. Neese, F. Wennmohs, A. Hansen, U. Becker, *Chem. Phys.* **2009**, *356*, 98; b) R. Izsák, F. Neese, *J. Chem. Phys.* **2011**, *135*, 144105.

[21] C. Angeli, R. Cimiraglia, S. Evangelisti, T. Leininger, J. P. Malrieu, *J. Chem. Phys.* **2001**, *114*, 10252.
